# An atlas of gross and histologic lesions and immunohistochemical immunoreactivity during the temporal progression of aerosolized Lassa virus induced hemorrhagic fever in cynomolgus macaques

**DOI:** 10.3389/fcimb.2024.1341891

**Published:** 2024-02-09

**Authors:** Forrest Bohler, Kathleen Cashman, Eric Wilkinson, Joshua C. Johnson, Kyle Rosenke, Josh Shamblin, Lisa Hensley, Anna Honko, Carl Shaia

**Affiliations:** ^1^ Laboratory of Virology, National Institute of Allergy and Infectious Diseases, Division of Intramural Research, National Institutes of Health (NIH), Hamilton, MT, United States; ^2^ Virology Division, United States Army Medical Research Institute of Infectious Diseases (USAMRIID), Frederick, MD, United States; ^3^ Zoonotic and Emerging Disease Research Unit, United States Department of Agriculture (USDA), Manhattan, KS, United States; ^4^ Rocky Mountain Veterinary Branch, National Institute of Allergy and Infectious Diseases, Division of Intramural Research, National Institutes of Health (NIH), Hamilton, MT, United States

**Keywords:** Lassa virus, Lassa fever, cynomolgus macaques, non-human primate, histologic lesion, immunohistochemistry

## Abstract

Lassa virus (LASV) causes an acute multisystemic hemorrhagic fever in humans known as Lassa fever, which is endemic in several African countries. This manuscript focuses on the progression of disease in cynomolgus macaques challenged with aerosolized LASV and serially sampled for the development and progression of gross and histopathologic lesions. Gross lesions were first noted in tissues on day 6 and persisted throughout day 12. Viremia and histologic lesions were first noted on day 6 commencing with the pulmonary system and hemolymphatic system and progressing at later time points to include all systems. Immunoreactivity to LASV antigen was first observed in the lungs of one macaque on day 3 and appeared localized to macrophages with an increase at later time points to include immunoreactivity in all organ systems. Additionally, this manuscript will serve as a detailed atlas of histopathologic lesions and disease progression for comparison to other animal models of aerosolized Arenaviral disease.

## 1 Introduction

Although it has been 54 years since Lassa fever was first recognized in Nigeria in 1969, a complete understanding of the pathology associated with Lassa virus (LASV) infection remains elusive, as does a specific treatment or vaccine. (Frame et al., 1970; Garry, 2023) Lassa fever remains endemic in the West African countries of Sierra Leone, Liberia, Nigeria, and Guinea where it is reported to infect approximately 100,000 to 300,000 people each year resulting in a severe and fatal hemorrhagic disease responsible for approximately 5,000 deaths annually. (McCormick and Fisher-Hoch, 2002; Lassa Fever | CDC, 2022) In addition to endemic Lassa fever in West Africa, there is currently an outbreak of Lassa fever in Nigeria dating back to 2019 with a case fatality rate hovering around 20%.(Nwafor et al., 2021) LASV is on the Health and Human Services Select Agents and Toxins list as it poses a severe threat to human health and is listed on the World Health Organizations list of priority pathogens with the potential to cause outbreaks or pandemics. (“Prioritizing Diseases for Research and Development in Emergency Contexts”, 2023; “HHS and USDA Select Agents and Toxins: 7 CFR Part 331, 9 CFR Part 121, and 42 CFR Part 73,” 2023) It is also classified as a Centers for Disease Control and Prevention Category A bioterrorism agent because it can be transmitted from person-to-person, can have a high mortality rate giving it the potential for a major public health impact, may cause panic and social disruption, and requires special action for public health preparedness. (CDC | Bioterrorism Agents/Diseases, 2019) The Federal Select Agent and the NIH Guidelines for Research Involving Recombinant or Synthetic Nucleic Acid Molecules (NIH Guidelines for Research Involving Recombinant or Synthetic Nucleic Acid Molecules (NIH Guidelines), 2019) list LASV in Risk Group 4 because it can cause serious or lethal human disease for which preventive or therapeutic interventions are not usually available, and it carries a high individual and community risk (Childs et al., 1995; Colangelo et al., 2013; Lassa Fever | CDC, 2022).

Lassa fever is a zoonotic disease transmitted by its natural reservoir the multimammate rat (*Mastomys natelensis*) through virus shed in the urine and feces, which is believed to become aerosolized and inhaled by the human victim and also potentially contracted through the improper preparation and consumption of the rodent as a food source. (Kouadio et al., 2015; Bonwitt et al., 2017) LASV belongs to the mammalian family of Old World Arenaviridae known as Mammarenaviridae (order Bunyavirales) which are small, enveloped, single-stranded RNA viruses. (Happi et al., 2019; Radoshitzky and de la Torre, 2019) The LASV genome is bisegmented with a large and small segment encoding for viral polymerase (L), a zinc finger motif protein (Z), viral nucleoprotein (NP), and the glycoprotein precursor (GPC) which is processed to form three functional subunits: the stable signal protein (SSP), Glycoprotein (GP) 1 and GP2. (Strecker et al., 2003; Hass et al., 2004; Cohen-Dvashi et al., 2016) Alpha-distroglycan has been identified as the cellular receptor for the LASV glycoprotein and is expressed on several human cell types including dendritic cells and vascular endothelial cells. (Cao et al., 1998; Kunz et al., 2005) In order for the virus to successfully infect host cells, it must first undergo a pH-dependent switch of its alpha-distroglycan receptor to the lysosome-associated membrane protein 1 (LAMP1) (Cohen-Dvashi et al., 2016).

The incubation period for LASV in humans ranges from 3 to 21 days with most patients becoming ill around day 7. (Richmond and Baglole, 2003) Many cases remain nonclinical and are self-limiting; however, when clinical signs and symptoms first occur, they include fever, sore throat, lethargy, dizziness, anorexia, retrosternal pain, lumbosacral pain, and myalgia. (Macher and Wolfe, 2006; Shehu et al., 2018) Patients may develop a maculopapular rash, nonpruritic and nonexudative conjunctivitis, pharyngitis, cough, and gastrointestinal symptoms including nausea, vomiting, diarrhea, epigastric discomfort, and intense abdominal pain. (Eze et al., 2014; Brosh-Nissimov, 2016; Kofman et al., 2019; Karnam et al., 2023) Edema may follow decreased blood pressure and increased vascular permeability followed by proteinuria, shock, hemorrhagic diathesis, a comatose state, and death. Survivors may develop neurologic symptoms such as confusion, tremors, ataxia, and seizures weeks to months later. (Günther et al., 2001; Okokhere et al., 2013) Tragically, up to 95% of pregnant women in their third trimester who are infected with LASV abort and/or succumb to the disease. (Price et al., 1988; Kayem et al., 2020) Also of great interest and impact, approximately 30% of surviving patients develop sudden-onset hearing loss. (Ficenec et al., 2020) Antibody mediated therapy and vaccine development continue with focus on the viral glycoprotein. (Cross et al., 2016; Mire et al., 2017; Cross et al., 2019; Brouwer et al., 2022) Although there are no current FDA-approved vaccines or therapeutics for the prevention or treatment of Lassa fever, in some studies the anti-viral Ribavirin has been shown to improve disease outcomes in both humans and non-human primates (NHPs) when administered early in the course of disease (Herring et al., 2021; Salam et al., 2022).

This manuscript describes the gross and histologic lesions of Lassa fever in cynomolgus macaques (*Macaca fascicularis*) when challenged by the aerosol route of exposure which is considered a natural route of exposure for the victims of Lassa fever. This study was designed to identify and describe the progression of clinical and tissue pathology during aerosolized LASV induced hemorrhagic fever in cynomolgus macaques through serial sacrifice at specified time points throughout the course of disease. A detailed description of the lesions associated with aerosolized Lassa fever in cynomolgus macaques adds to the understanding of the disease, it’s symptomology, and allows for improved comparison to humans suffering from Lassa fever. Additional benefits include a detailed tool for comparing lesions from other animal models of Lassa fever such as the well described Strain 13 guinea pig model, a comparison to the hemorrhagic fevers caused by other Old and New World arenaviruses, and lesions associated with other hemorrhagic fever virus families such as Filoviridae. Specifically, this report is intended to detail the distribution and progression of gross and histologic lesions, as well as anti-LASV immunoreactivity via IHC, associated with an aerosol exposure to LASV from day one through day 12 post-exposure (PE) and to demonstrate and describe with photomicrographs the wide range of tissues affected by this virus.

## 2 Materials and methods

### 2.1 Ethics statement

Research was conducted under a USAMRIID Institutional Animal Care and Use Committee approved protocol in compliance with the Animal Welfare Act, Public Health Service Policy on Humane Care and Use of Laboratory Animals, and other federal statutes and regulations relating to animals and experiments involving animals. The facility where this research was conducted is accredited by the Association for Assessment and Accreditation of Laboratory Animal Care International and adheres to principles stated in the Guide for the Care and Use of Laboratory Animals, National Research Council, 1996.

### 2.2 Lassa virus exposure

Nineteen cynomolgus macaques were divided into 6 groups and exposed to a target dose of 1000 plaque forming units (PFU) of aerosolized LASV, Josiah strain. Each macaque was evaluated in a whole-body plethysmography box (Buxco Research Systems, Wilmington, NC, USA) to determine individual minute volume from which a challenge dose was calculated. Aerosolization was performed in a Class III biosafety cabinet inside a biosafety level-4 (BSL-4) laboratory using a 3-jet collision nebulizer (BGI Inc., Waltham, MA, USA) and with the head only automated bioaerosol exposure II (ABES-II) system developed at the United States Army Medical Research Institute of Infectious Diseases (USAMRIID). Plaque assays were performed on the starting concentration of the virus-containing dilutions prior to exposure and on the sample collected from the all glass impinger after each aerosol exposure run.

### 2.3 Detection of viral RNA by RT-PCR

Serum was also collected at each time point to measure viremia. RNA was isolated from the serum of LASV exposed macaques and prepared with TRI Reagent LS (Sigma-Aldrich). The aqueous phase was extracted using Phase Lock heavy gel tubes (5 Prime) and 1-Bromo-3-chloropane (BCP, Sigma-Aldrich) and mixed with 70% ethanol. Following 5 minutes of incubation at room temperature it was added to an RNeasy column and extracted according to manufacturer’s recommendations (Qiagen). RNA was eluted through two consecutive additions of 50ul of nuclease-free water and stored at -80°C until analysis. One-step quantitative real-time RT-PCR reactions were performed on a LightCycler 480 (Roche, Indianapolis, IN, USA) in 20ul volumes with 5ul of purified RNA and Superscript II One-Step RT-PCR system (Life Technologies). Primers and probe were specific for the LASV GP gene [Forward, 900nM: TgCTAgTACAgACAgTgCAATgAg; Reverse, 900 nM: TAgTgACATTCTTCCAggAAgTgC (Oligos Etc., Wilsonville, OR); Probe, 200 nM: TgTTCATCACCTCTTCMGBNFQ (Applied Biosystems)]. Cycling conditions were reverse transcription at 50°C for 15 minutes, and denaturation at 95°C for 5 minutes; then 45 cycles of 95°C for 1 second, 60°C for 20 seconds followed by a single acquisition, and a final cooling step of 40°C for 30 seconds. Absolute quantification was compared to a viral RNA standard using LC480 software (version 1.5.0.39) and a standard calibrator on each plate. We present viremia data as PFU equivalents/ml using a 10:1 PCR genome equivalent:PFU ratio that has been previously determined and validated on human LASV samples (Malhotra et al., 2013).

### 2.4 Necropsy, histopathology, and immunohistochemistry

Macaques were euthanized in accordance with AVMA guidelines on 1, 3, 6, 10, 11 and 12 days post exposure. Immediately following euthanasia, necropsies were performed in the USAMRIID animal BSL-4 laboratory. Tissues from all major organ systems were collected for histopathological examination and immersion fixed in 10% neutral buffered formalin for a minimum of 21 days. Fixed tissues were trimmed, embedded, sectioned, and stained with hematoxylin and eosin (H&E) in accordance with institute protocol. LASV IHC was performed on replicate tissue sections using an Envision-PO kit. Slide preparation and processing was then followed using the protocol outlined in Bell, et al. (Bell et al., 2017).

LASV antigen was semi-quantitatively documented using a scale of 1 to 4, with 1 representing rare (1-10%) staining; 2 occasional (11-25%); 3 frequent (26-50%) and 4 abundant (>50%). A board-certified veterinary pathologist evaluated the H&E and IHC slides using a Nikon Eclipse 80i light microscope.

## 3 Results and discussion

**Figure 1:**
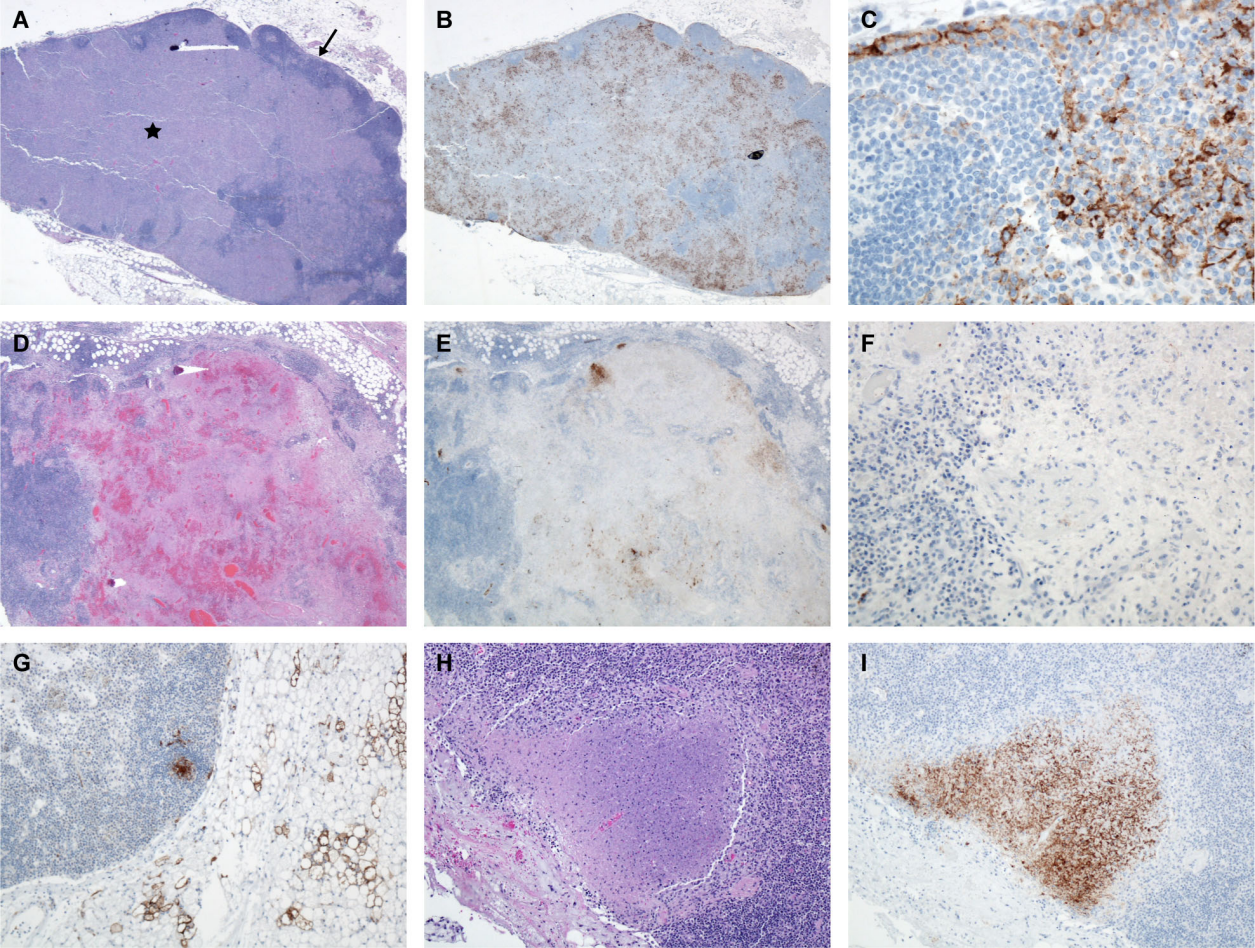
Tracheobronchial LN, Monkey 9, Day 6 PE. **(A)** Note the inner cortex and medulla is obscured by fibrin, edema and mononuclear cells (*) with a thin rim of outer cortex composed of lymphoid follicles (➔) H&E, 20x. **(B)** Immunoreactive mononuclear cells throughout the inner cortex and medulla, LASV IHC, 20x. **(C)** Higher magnification demonstrating immunoreactive mononuclear cells (dendritic cells, macrophages) throughout the subcapsular sinus and parafollicular cortex, LASV IHC, 400x. Tracheobronchial LN, Monkey 11, Day 10 PE. **(D)** Necrosis, hemorrhage, fibrin, and edema obscure the inner cortex and medulla, H&E, 20x. **(E)** There are a few clusters of immunoreactive cells present, LASV IHC, 20x. **(F)** Higher magnification demonstrating paucity of immunoreactive cells, LASV IHC, 200x. Tracheobronchial LN, Monkey 16. Day 11 PE. **(G)** Immunoreactive cells in center of follicle and perinodal adipocytes. LASV IHC, 100x. Tracheobronchial LN, Monkey 18, Day 12 PE. **(H)** Focus of necrosis at periphery of node, H&E, 100x. **(I)** Immunoreactivity in adjacent focus of necrosis and absent in the rest of the tissue, LASV IHC, 100x.

**Figure 2:**
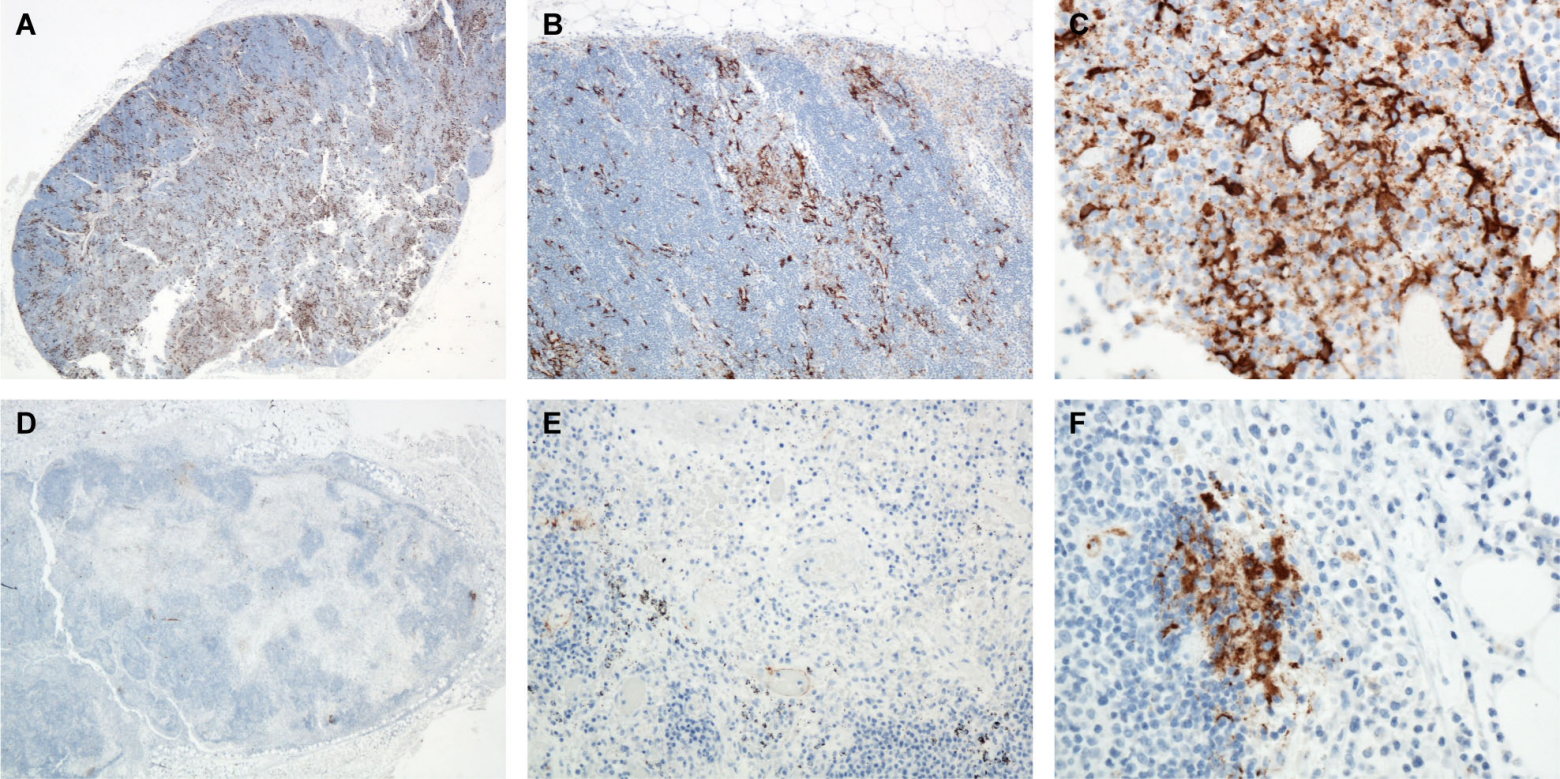
Mediastinal LN, Monkey 8, Day 6 PE. **(A)** 20x, **(B)** 100x, **(C)** 400x. Immunoreactive mononuclear cells throughout the sinuses. LASV IHC. Mediastinal LN, Monkey 11, Day 10 PE. LASV IHC. **(D)** 20x, **(E)** 200x, **(F)** 400x. Note the marked reduction in immunoreactive cells by day 10 PE with only a few clusters of immunoreactive cells present.

A total of nineteen cynomolgus macaques were exposed to LASV Josiah by the aerosol route. Animals were divided into six groups of three each (except day 6, which was a group of 4 animals with 2 challenged during the first exposure and 2 during the second), and were serially euthanized at days 1, 3, 6, 10, 11, and 12 PE. There was variation in lesion distribution and severity between individual animals and between groups. In general; however, the macaques displayed a linear increase in serum viral genomic material, histologic lesions and immunohistochemical immunoreactivity the longer they survived. This finding comes with the exception of the mediastinal and tracheobronchial lymph nodes, which had greatly reduced numbers of immunoreactive cells on day 10 PE (**Figures 1**, **2**).

### 3.1 Viral genomes in serum

**Figure 3:**
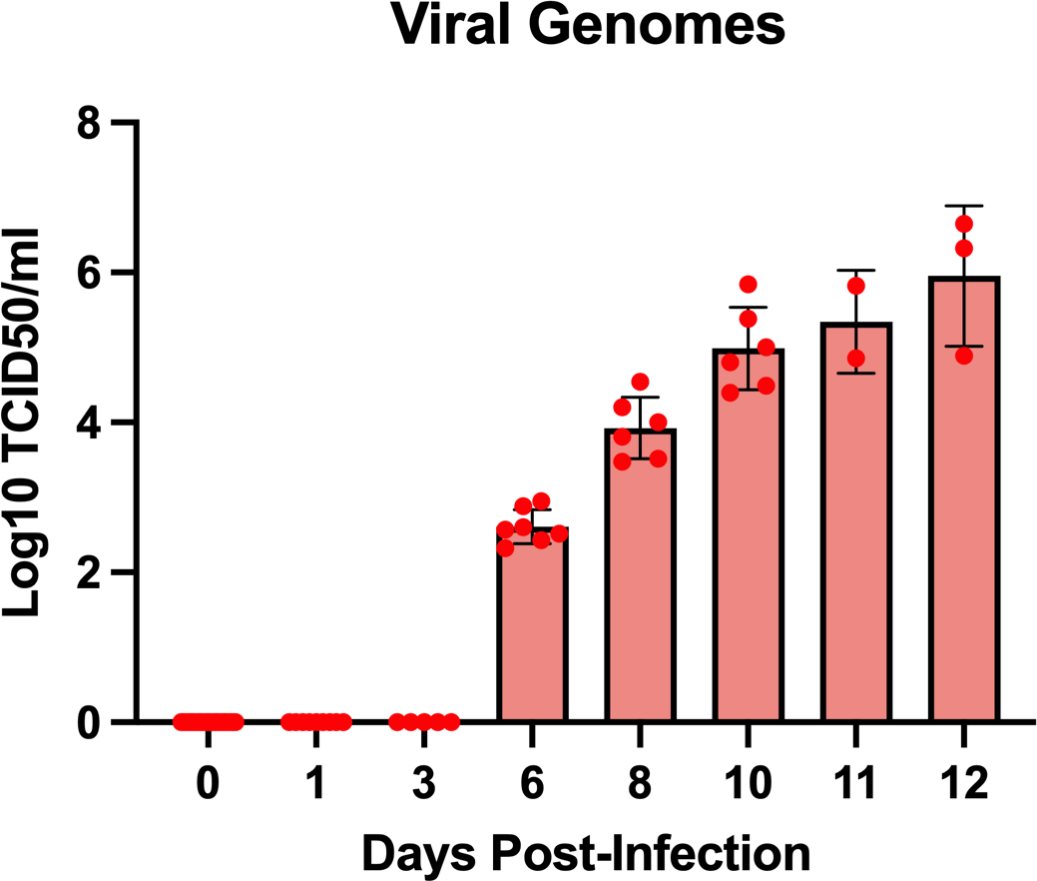
Presence of Lassa viral genomes in serum detected by qRT-PCR.

Lassa viral genomic material was first detected in serum via qRT-PCR on day 6 post exposure. Viral genome copy numbers continued to increase throughout the remainder of the study (**Figure 3**).

### 3.2 Gross pathology

**Figure 4:**
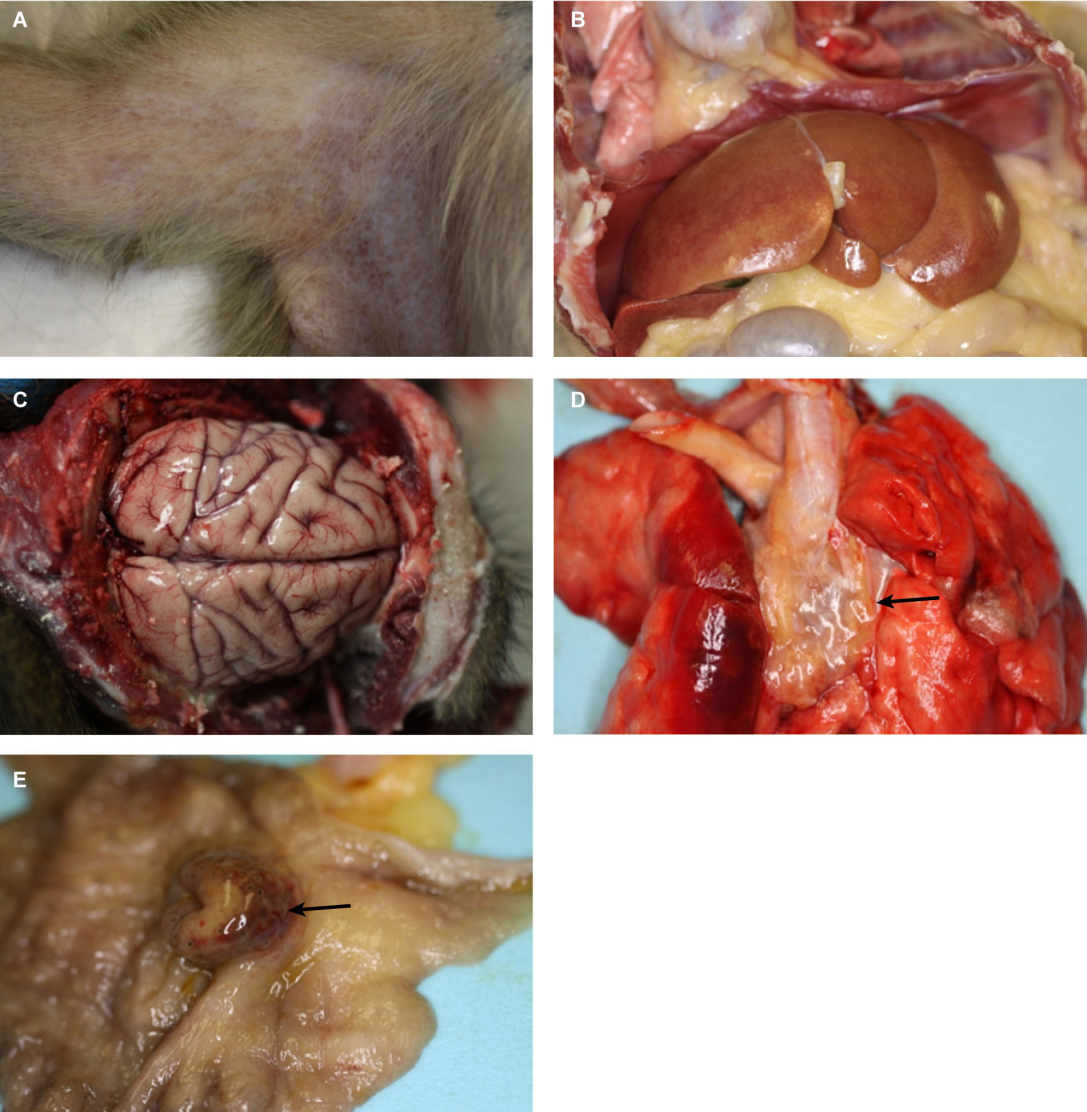
Gross pathologic findings, **(A)** Axillary rash. Monkey 18 Day 12 PE. **(B)** Enlarged pale liver. Monkey 12 Day 10 PE. **(C)** Congested and cloudy meninges. Monkey 14 Day 11 PE. **(D)** Hyperemic lungs with edema at the level of the tracheobronchial lymph nodes (Arrow). Monkey 19 Day 12 PE; **(E)** Mucosal hemorrhages and edema of the ileocecal junction (Arrow). Monkey 19 Day 12 PE.

Significant gross pathology noted at necropsy included dry and tacky subcutaneous tissues attributed to dehydration; dark, red lungs that failed to collapse completely when the thorax was opened; tracheobronchial and mediastinal lymph node enlargement; slightly enlarged and pale liver; slightly enlarged spleen; variable amounts of abdominal effusion; urinary bladder mucosal petechia; subcutaneous edema; and cloudy, congested meninges. Other findings included axillary and inguinal lymph node enlargement and cutaneous rash. In general, gross pathologic findings increased with severity over time. Specific gross lesions at day 6 PE included red and edematous tracheobronchial lymph nodes and the spleen in about half of the macaques was enlarged and firm with plump round edges. The liver was pale and swollen in day 6, 10, 11 and 12 PE macaques. By day 10 and 11 PE most macaques had subtle lung lesions such as redness, a failure to collapse completely upon opening the thorax, and variable amounts of effusion. Although pleural effusion existed in several of the day 10 through day 12 PE macaques, this finding was complicated by the intra-cardiac method of euthanasia, which often contaminated the thorax with blood. The pleural adhesions noted in 3 macaques were mature and fibrous suggesting a preexisting condition and were interpreted as having developed from a prior lung injury unrelated to the experimental protocol. The presence of cloudy (edematous) and congested meninges was also subtle yet consistent in the day 10, 11, and 12 PE macaques. A cutaneous rash on the face and axilla was not observed until day 12 PE in two out of three macaques. Petechia on the mucosa of the urinary bladder occurred in one of three of the day 11 and day 12 PE macaques, and petechia on the mucosa of the ileocecal junction occurred in two of the three day 12 PE macaques (**Figure 4**). Red nodules on the serosa and mucosa of the colon, believed to correlate with gut associated lymphoid tissue (GALT), occurred in one of three of the day 11 and 12 PE macaques. Peri-testicular, scrotal, and/or inguinal sub-cutaneous edema was present in one of the three day 10 and day 11 PE macaques. To summarize, cynomolgus macaques demonstrated a progression of gross lesions from day 6 through day 12 PE (**Table 1**).

**Table 1:** Summary of gross pathology noted at necropsy.

Animal	Day necropsied post-exposure	Sex	Rash, face	Rash, body	Lungs	Spleen	Liver	Meninges	Mediastinal & Tracheobronchial Lymph Nodes	Peripheral Lymph Node	Mesenteric Lymph Node
Monkey 1	1	M	No	No	No	No	No	No	No	No	No
Monkey 2		M	No	No	No	No	No	No	No	No	No
Monkey 3		M	No	No	No	No	No	No	No	No	No
Monkey 4	3	M	No	No	No	No	No	No	No	No	No
Monkey 5		F	No	No	No	No	No	No	No	No	No
Monkey 6		M	No	No	No	No	No	No	No	No	No
Monkey 7	6	M	No	No	No	S	P, S	WC	R, S	No	No
Monkey 8		M	No	No	PA	S	P, S	No	R, S	No	No
Monkey 9		F	No	No	No	No	No	No	No	No	No
Monkey 10		F	No	No	No	No	No	No	No	No	No
Monkey 11	10	M	No	No	Ef	S	P	WC	R, S	No	S
Monkey 12		F	No	No	PA, Ef	No	P, S	WC	S	No	No
Monkey 13		M	No	No	No	S	P	WC	No	No	No
Monkey 14	11	M	No	No	No	S	P, S	WC	S, E	No	No
Monkey 15		M	No	No	R	No	P, S	WC	No	No	No
Monkey 16		M	No	No	R, Ef, PA	No	No	WC	S, E	No	No
Monkey 17	12	M	No	No	Ef	S	P, S	WC	No	No	No
Monkey 18		F	Yes	Yes	R, Ef	No	P, S	WC	S	No	No
Monkey 19		F	No	Yes	No	No	P, S	WC	E	R, S	R, S

Med, Mediastinal lymph node; TB lymph node, tracheobronchial lymph node; Mes lymph node, mesenteric lymph node; Peripheral lymph node, Axillary or Inguinal lymph node; R, red (congestion and hemorrhage); P, pale; E, edema; Ef, Effusion; S, size increase; N, necrotic; W within normal limits; PA, pleural adhesions to chest wall, likely previous, healed injury or infection; WC, wet and cloudy; F, female; M, male.

### 3.3 Histopathology and immunohistochemistry

#### 3.3.1 Lymphoid tissues

**Figure 5:**
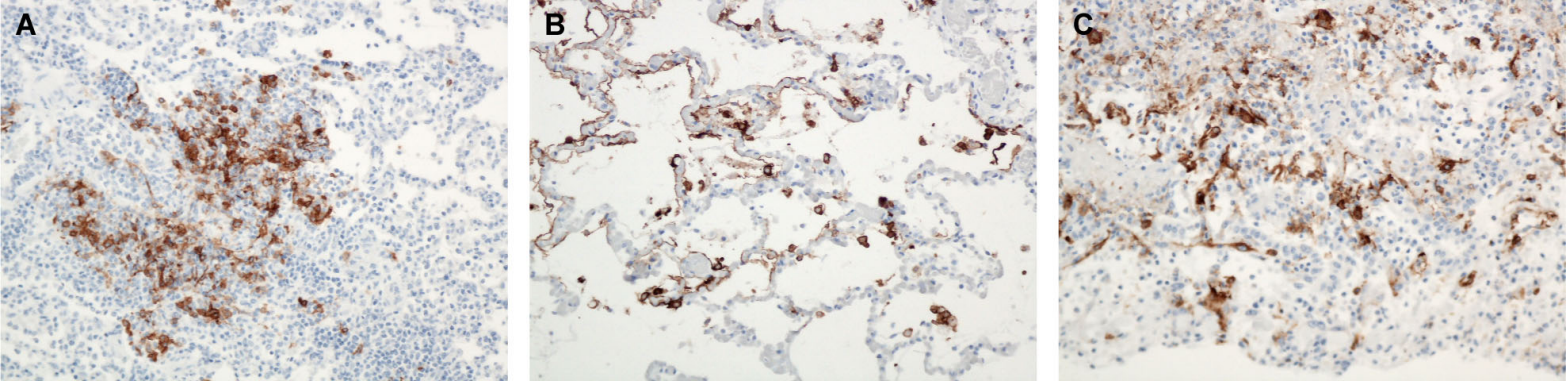
**(A)** Lung, Monkey 6, Day 3 PE. Immunoreactive mononuclear cells within a focus of inflammation. LASV IHC, 200x. **(B)** Lung, Monkey 8, Day 6 PE. Immunoreactive mononuclear cells and pneumocytes within a focus of slight interstitial pneumonia. LASV IHC, 200x. **(C)** Lung, Monkey 12, Day 10 PE. Immunoreactive mononuclear cells and pneumocytes within a focus of pleural inflammation and interstitial pneumonia. LASV IHC, 200x.

Lesions occurring within the tracheobronchial and mediastinal lymph nodes at day 6 PE included congestion, edema, fibrin, hemorrhage, variable amounts of necrosis/apoptosis of lymphocytes (lymphocytolysis), sinus histiocytosis, vessels lined by reactive endothelium, and generalized lymphoid depletion (**Figure 1**). High endothelial venules (HEVs) contained many mononuclear inflammatory cells or had vessel walls blurred by fibrin and edema. Mononuclear cells throughout the subcapsular and medullary sinuses were strongly immunoreactive as were numerous stellate mononuclear cells scattered throughout the nodes (dendritic cells). Two of the three day 10 PE macaques had coalescing foci of necrosis with hemorrhage, fibrin, edema and vasculitis. There was often scattered necrotic/apoptotic cellular debris within the hemorrhagic foci which demonstrated immunoreactivity; however, the overall prevalence of immunoreactivity was dramatically reduced compared to the day 6 PE nodes. Following the highest observed level of immunoreactivity at day 6 PE in numerous mononuclear cells (macrophages and dendritic cells), the remainder of the time points had dramatically reduced numbers of immunoreactive cells with persistent staining only in scattered endothelial cells, scattered and rare mononuclear cells (macrophages and dendritic cells), extracellular debris in germinal centers, and scattered debris throughout the nodes. Endothelial cells demonstrated immunoreactivity beginning on day 6 PE and persisted throughout the remainder of the timepoints, generally in progressively decreasing numbers. Although still present in the day 11 and day 12 PE macaques, lesion severity appeared to dramatically decrease as well (**Figures 4**, **5**). These findings are consistent with an aerosol challenge as tracheobronchial and mediastinal lymph nodes receive lymph from the pulmonary tract and may serve as the source of lymphatic spread to other lymph nodes and the spleen. The remainder of the peripheral lymph nodes developed lesions less severe than the tracheobronchial and mediastinal lymph nodes which did not manifest until day 10 PE; however, by day 10 PE, multiple lymph nodes demonstrated a profound sinus histiocytosis, congestion, edema, variable amounts of hemorrhage and lymphoid depletion.

#### 3.3.2 Lungs

**Figure 6:**
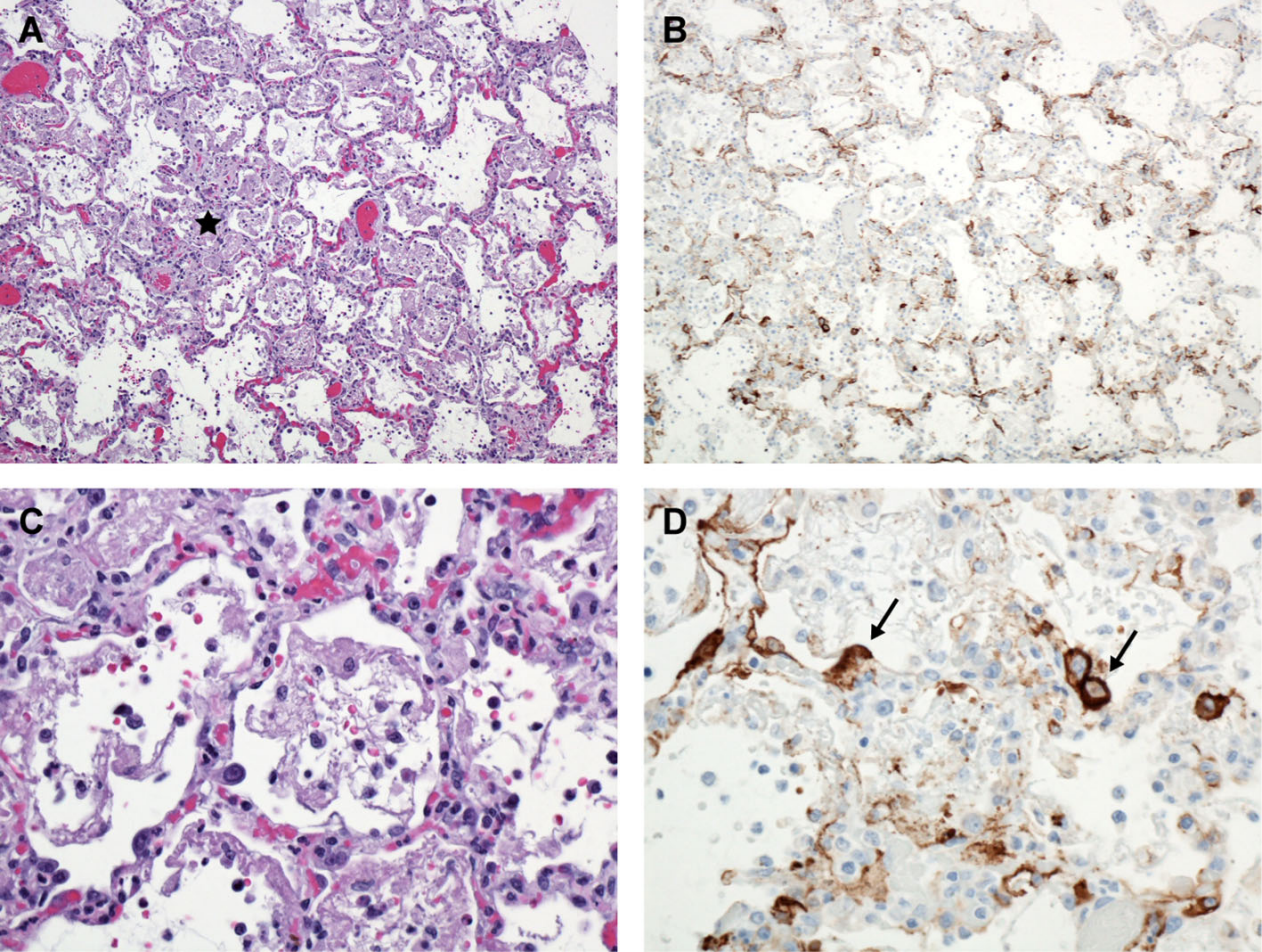
Lung, Monkey 16. Day 11 PE. **(A)** Alveolitis and interstitial pneumonia characterized by thickened alveolar septa, sub-acute inflammation, alveolar fibrin, and edema (*). H&E, 100X. **(B)** Numerous immunoreactive mononuclear cells presumed to be alveolar macrophages and occasional pneumocytes; LASV IHC, 100X. **(C)** Higher magnification, demonstrating alveolar macrophages, fibrin, and mildly thickened alveolar septa. H&E, 400X. **(D)** Higher magnification, demonstrating immunoreactive alveolar macrophages and pneumocytes (➔). LASV IHC, 400X.

Subtle histologic lung lesions attributable to LASV first appeared at day 3 PE, increased by day 6 PE, and resulted in interstitial pneumonia in seven out of nine monkeys by days 10, 11, and 12 PE. Some peracute histologic changes in the lungs were due in part to intra-cardiac administration of euthanasia solution; however, at later timepoints foci of alveolitis and interstitial pneumonia also demonstrated immunoreactivity. Specifically, perivascular edema present in one of the day 1 PE macaques was attributed to the method of euthanasia. Early inflammation in the lungs may be due to aerosol exposure and only appeared after three days; whereas, alveolitis and interstitial pneumonia, although not a diffuse process, increased with time, as did immunoreactivity in foci of inflammation. Specific changes of alveolitis were first present in one of the day 3 PE macaques and two of the four day 6 PE macaques which were similarly affected. By day 10 PE all the macaques had both evidence of alveolitis and mild interstitial pneumonia. Inflammation was predominantly histiocytic and partially filled alveoli, expanded alveolar septa and was mixed with edema and fibrin. More severe lesions consisted of foci of necrotic pneumocytes, cellular debris and occasional syncytial cells. Multifocally, interstitial and alveolar macrophages and fewer type II pneumocytes exhibited cytoplasmic immunoreactivity, as did rare small patches of bronchial epithelial cells. Additionally, endothelial cells multifocally exhibited immunoreactivity, as did reactive mesothelial cells (**Figure 6**).

#### 3.3.3 Spleen

**Figure 7:**
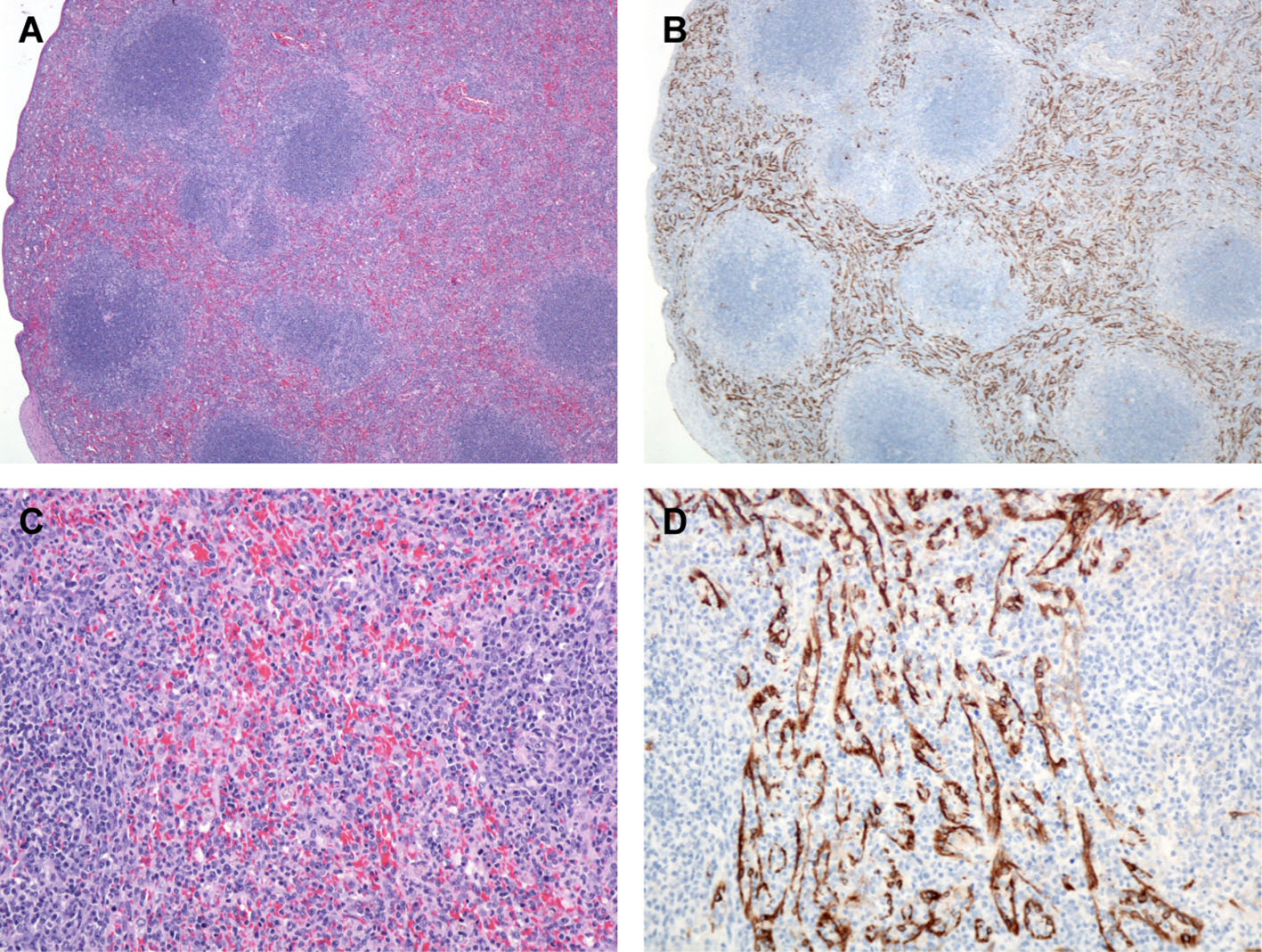
Spleen, Monkey 17 Day 12 PE. **(A)** The red pulp is expanded by numerous histiocytes. H&E, 40X. **(B)** Numerous immunoreactive mononuclear cells presumed to be venous sinus endothelial cells and FRCs. LASV IHC, 40X. **(C)** Higher magnification demonstrating numerous plump mononuclear cells throughout the red pulp. H&E, 200X. **(D)** Higher magnification, demonstrating immunoreactive venous sinus endothelial cells and FRCs. LASV IHC, 400X.

Fibrin and edema within the red pulp of the spleen were present at day 6 PE, as well as days 10, 11, and 12 PE. On days 10, 11, and 12 there were increased mononuclear cells (histiocytes and lymphocytes) and fewer neutrophils throughout the red pulp with or without mononuclear vasculitis characterized by disrupted vascular tunics predominantly in medium-sized veins with sub-intimal and transmural histiocytes and lymphocytes (**Figure 7**). Determining the role of neutrophils in the splenic inflammation was difficult without complete blood counts to corroborate leukocytosis. In the white pulp, there was occasional follicular germinal center necrotic/apoptotic debris. Immunoreactive sinus endothelial cells and reticular cells were outlined by the immunostain and present diffusely throughout the red pulp where they appeared as linear vessels lined by immunoreactive cells. There was also minimal to mild lymphoid follicle germinal center mononuclear cell cytoplasmic immunoreactivity and immunoreactive extracellular debris (**Figure 7**). Lymphoid hyperplasia within the spleen and lymph nodes in several animals at various timepoints was likely a response to antigenic stimulation either prior to the start or during this study.

#### 3.3.4 Liver

**Figure 8:**
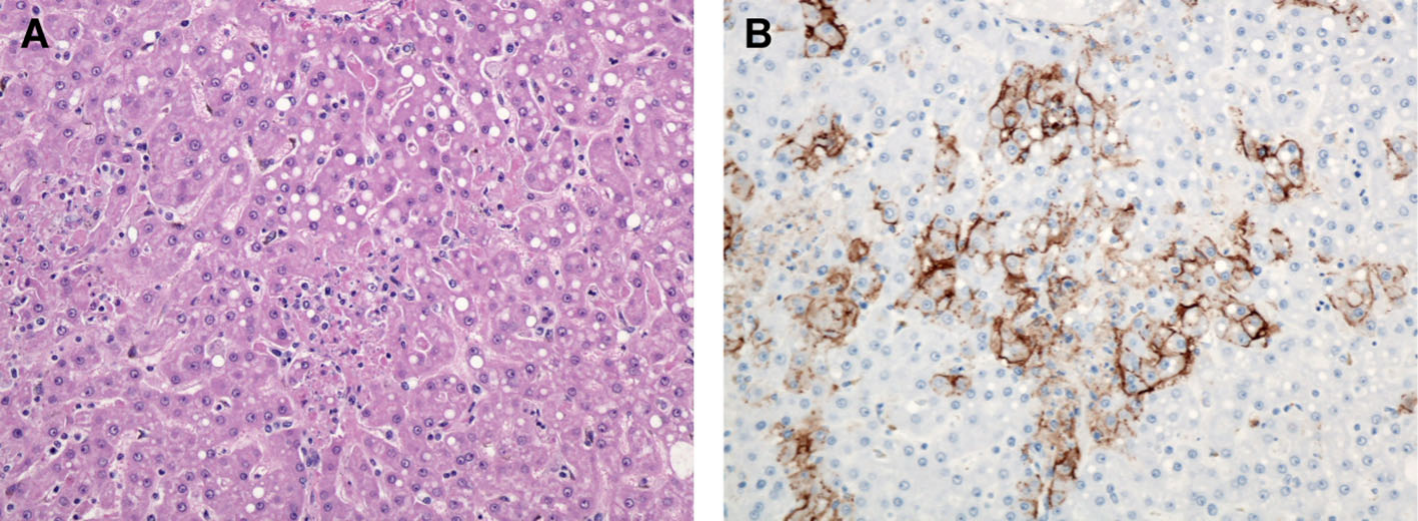
Liver, Monkey 18. Day 12 PE. **(A)** Multiple foci of hepatocellular necrosis with mild lipid-type vacuolar degeneration. H&E, 400X. **(B)** Multifocal immunoreactive hepatocytes and mononuclear cells presumed to be Kupffer cells demonstrating predominantly membrane associated immunoreactivity. LASV IHC, 400X.

Hepatic lesions included increased inflammatory cells within the sinusoids and apoptotic/necrotic Kupffer cell at days 10, 11, and 12 PE. Hepatic necrosis occurred in only one of three day 12 PE macaques in this study and was randomly distributed and often distended sinusoids with cell debris (**Figure 8**). This necrosis was later in onset when compared to other work challenging cynomolgus macaques via the intramuscular (IM) route in which hepatic necrosis was observed as early as day 7 PE (Hensley et al., 2011). Neutrophilic inflammation was sparse; however, sinusoids diffusely contained more inflammatory cells than normal. Oftentimes foci of inflammation contained necrotic single cell debris and adjacent hepatocytes exhibited strong cytoplasmic and membrane associated immunoreactivity. Mild portal edema and/or ectatic lymphatic vessels were also noted in the day 10, 11 and 12 PE macaques and lipid-type vacuolar degeneration was minimal.

#### 3.3.5 Brain

**Figure 9:**
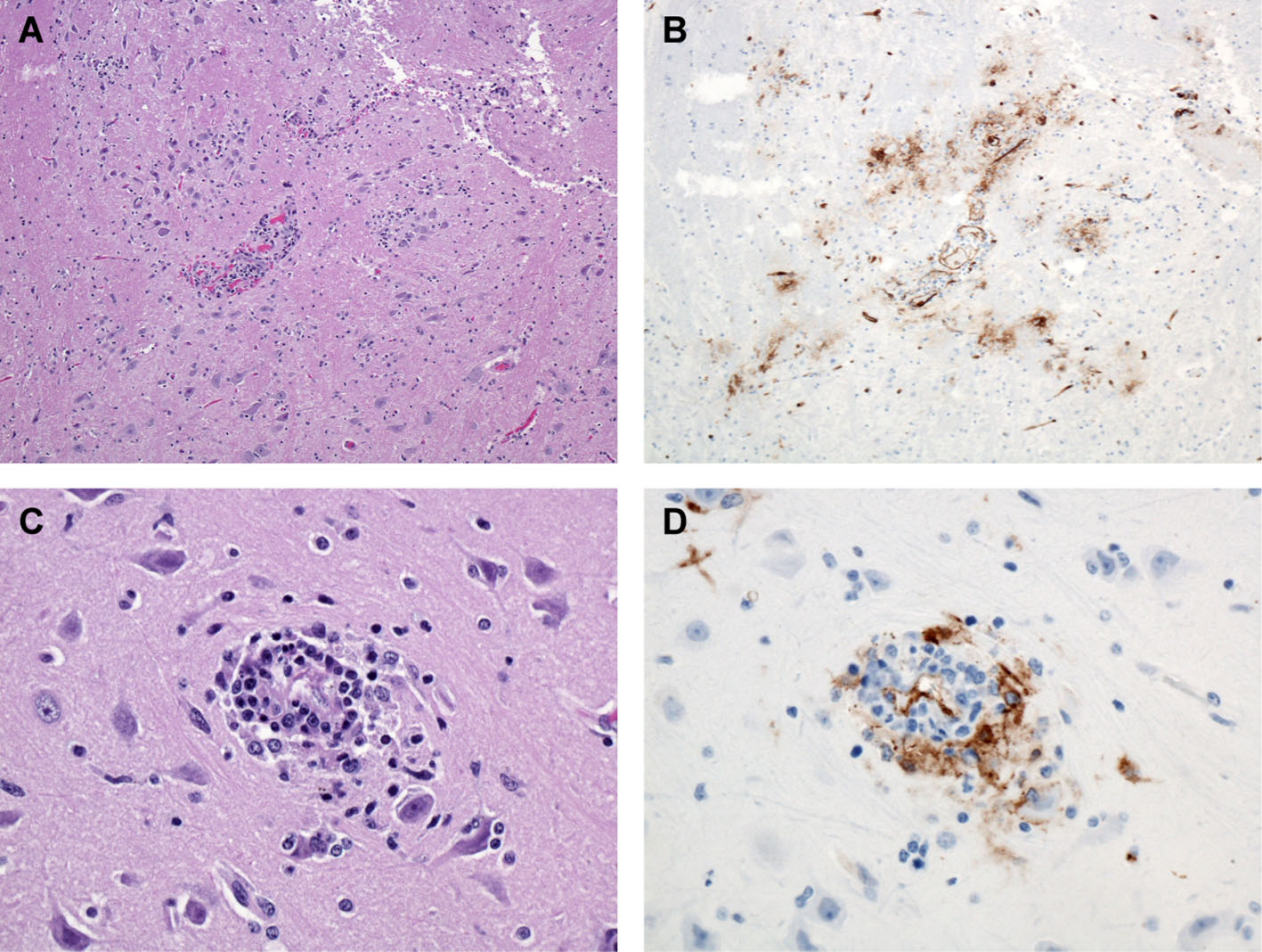
Brain, Monkey 15. Day 11 PE. **(A)** Mononuclear inflammation surrounding blood vessels and scattered locally throughout the section. H&E, 100X. **(B)** Numerous immunoreactive blood vessels and immunoreactive debris within the adjacent tissue. LASV IHC, 100X. **(C)** Higher magnification, demonstrating small focus of perivascular mononuclear (lymphocytes and macrophages) inflammation and adjacent neuronal satellitosis. H&E, 400X. **(D)** Higher magnification, demonstrating immunoreactivity associated with the vessel and surrounding inflammatory cells. LASV IHC, 400X.

Histologic lesions in the brain were subtle yet correlated directly with the gross observations of congested blood vessels and wet or cloudy meninges (edema). In three out of six of the day 11 and day 12 PE macaques there was perivascular hemorrhage, edema, and inflammation within the meninges, Virchow-Robin spaces, choroid plexus, and pituitary gland with occasional extension into the adjacent neuropil. Inflammation consisted of lymphocytes and histiocytes contributing to perivascular cuffing as well as vessels lined by marginating leukocytes and reactive endothelial cells. In rare cases there was satellitosis associated with neurons adjacent to affected vessels. These foci of inflammation were often immunoreactive, as were capillary and larger vessel endothelial cells throughout the brain and meninges (**Figure 9**). Unlike a similar study that investigated the natural history of aerosolized LASV in macaques, in which necrosis of the neuropil was noted, none of the serial sacrifice macaques developed obvious foci of necrosis within the neuropil within our time points (Downs et al., 2020).

#### 3.3.6 Kidney and adrenal glands

Lesions in the kidney appeared on day 10 PE and included perivascular edema and interstitial inflammation. On day 11 and day 12 PE lesions of the adrenal glands were limited to degeneration, scattered single cell and small clusters of necrosis, congestion, and increased interstitial cellularity attributed to increased circulating inflammatory cells throughout the cortical capillaries. Immunoreactivity was widespread with adrenal cortical cells exhibiting multifocal membrane associated and, less often, cytoplasmic immunoreactivity present on day 10 PE and widespread by day 11 and day 12 PE with a relative sparing of the medulla (see **Supplementary Figure S1**).

#### 3.3.7 Other tissues and organs

By day 10 and 11 PE, epithelial cells in a variety of tissues were undergoing necrosis, generally noted in small clusters of cells and a relative paucity of associated inflammation (see **Supplementary Figures S1–S12**). The presence of in numerous epithelial lined organs with little to no inflammation is of great interest. Organs and tissues demonstrating single cell and small clusters of necrotic cells included the tongue, esophagus, larynx, tonsillar epithelium, renal pelvis, urinary bladder, and epididymis. Readers are encouraged to review the Supplement where photomicrographs of several tissues and organs with lesions and/or immunoreactivity are reported, to our knowledge, for the first time and include the tongue, esophagus, gastric mucosa, laryngeal epithelium, colon, parathyroid gland, pancreatic islets of Langerhans cells, conjunctiva of the eye, penile urethra, ovarian and uterine stroma and epididymis. In nearly all cases, these lesions demonstrated LASV immunoreactivity. Of interest, immunoreactivity was noted in the ovarian stroma of one of two of the female macaques from day 6 PE (see **Supplementary Figure S10**).

A complete listing of the histology results are summarized in **Supplementary Table 1** and IHC results are summarized in **Supplementary Table 2**.

### 3.4 Lassa virus antigen immunohistochemistry

Initially, immunoreactivity appeared in alveolar macrophages in one of three day 3 PE macaques and two of four day 6 PE macaques. The immunoreactive cells were located within foci of alveolar inflammation (alveolitis), and there is some speculation that these foci of inflammation preexisted and predated the aerosol exposure. Regardless, alveolar macrophages were immunoreactive and were the first cells to demonstrate immunoreactivity following aerosol exposure. Although the number of immunoreactive cells (alveolar macrophages and pneumocytes) increased over time, neither alveolitis and interstitial pneumonia nor the presence of immunoreactive cells progressed to a diffuse process and within the lung sections of each macaque there were areas of normal and non-immunoreactive tissue (**Figure 5**).

**Figure 10:**
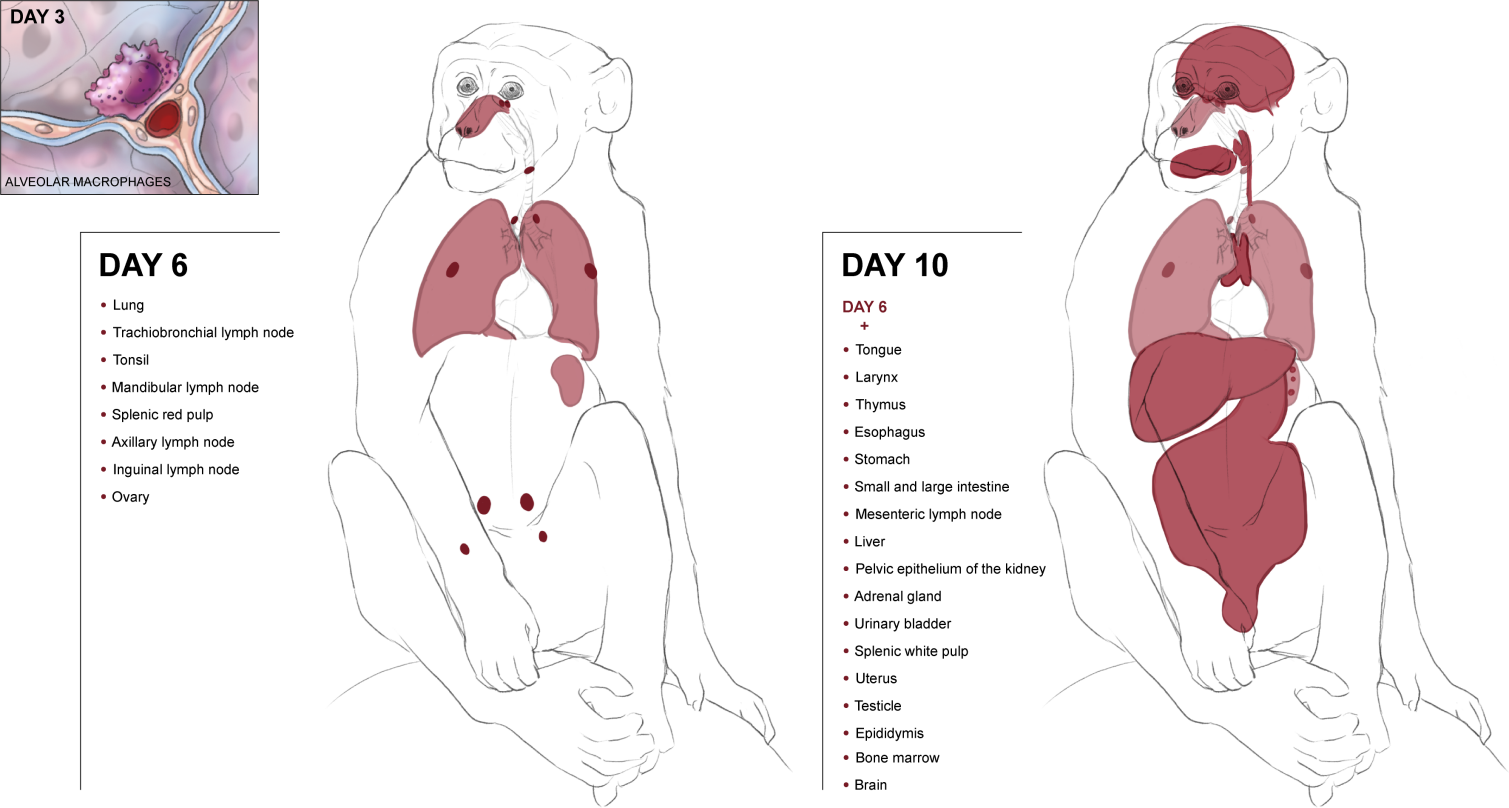
Temporal detection of viral antigen.

By day 6 PE, there were multiple foci of immunoreactive mononuclear cells (macrophages) within several sampled lymph nodes as well as the spleen with the highest number occupying the sinuses of the mediastinal and tracheobronchial lymph nodes followed by sinus endothelial cells and fibroblastic reticular cells. On day 10 PE, immunoreactivity was widespread and in multiple tissues including the remaining lymph nodes, intra-sinusoidal hepatic stellate (Kupffer) cells and small clusters of hepatocytes (see **Supplementary Figures S13**, **S20–S23**). Simply stated, following aerosol exposure and viral entry, anti-LASV immunoreactivity first appeared in day 3 alveolar macrophages, was especially prominent in the tracheobronchial and mediastinal lymph nodes by day 6 PE as well as other lymphoid organs in lesser amounts, and then appeared systemic by day 10 (**Figure 10**).

Of note, smudgy intracytoplasmic basophilic inclusion bodies were often noted in mononuclear cells of the spleen and lymph nodes as early as day 6. TEM demonstrated the presence of intracytoplasmic inclusion bodies in multiple cell types to include macrophages and endothelial cells, which are believed to represent interferon production by the host cell. By TEM, these inclusion bodies appeared as well-organized paracrystalline arrays, and their presence is well-documented in non-human primates with viral disease (1-4).

*En bloc* sections of the external, middle, and inner ear were removed from the skull at necropsy. Our intention was to identify lesions in the ear which could correlate to the clinical observation of deafness noted in some human patients who have recovered from Lassa fever (White, 1972). Although histologic lesions in the ear were not identified, there was immunoreactivity noted in the vestibulocochlear nerve that innervates the ear and is responsible for the transmission of sound signals to the brain. Immunoreactivity was often associated with neurons and the fibrovascular tissue within the nerve (**Supplementary Table 2**; **Supplementary Figures S18**, **S19**). Although the pathogenesis of hearing loss has not been completely elucidated, early studies have been published which suggested an immune response rather than direct viral damage as being responsible (Cummins et al., 1990); and more recent work supports that hypothesis by demonstrating an immune-mediated systemic vasculitis as a contributing factor (Cashman et al., 2018).

There is much room for continued research within this study, and additional studies are needed to further characterize the progression of disease. Additional IHC with antibodies specific for macrophages, follicular and interdigitating dendritic cells would further characterize the contributions of these cellular targets in the progression of disease especially and specifically within sections of mediastinal lymph node from day 3 through 10 PE macaques. Furthermore, *in situ* hybridization (ISH) could reveal the presence of the viral genome and possibly replicating virus in the same catalog of tissues. Additionally, sections of mediastinal and tracheobronchial lymph node, spleen, lung, adrenal gland, and liver should be prepared for transmission electron micrography (TEM) with the intent of identifying Lassa virions in tissue. The profound immunoreactivity of the venous sinuses of the spleen (**Figure 7**) and vessels systemically suggests involvement of endothelial cells and additional studies should be pursued to better characterize their role in the progression of disease.

Single cell necrosis and small clusters of necrotic cells in the epithelium of multiple tissue types (e.g. gastrointestinal, urinary, and reproductive systems) with corresponding specific anti-LASV immunoreactivity leads to speculation on the methods of viral shedding and transmission.

Virus isolation from a host of these tissues will need to be performed to determine the presence of viable virus. Additional *in situ* hybridization studies should be performed to demonstrate the presence or absence of replicating virus, which could be shed from these epithelial cells into feces, urine, and other genitourinary fluids. Collection of these samples and further analysis on the presence or absence of replicating virus may contribute to further understanding of how LASV is shed and transmitted between individuals.

## Data availability statement

The original contributions presented in the study are included in the article/**Supplementary Material**. Further inquiries can be directed to the corresponding author.

## Ethics statement

The animal study was approved by USAMRIID Institutional Animal Care and Use Committee. The study was conducted in accordance with the local legislation and institutional requirements.

## Author contributions

FB: Data curation, Formal analysis, Investigation, Visualization, Writing – original draft, Writing – review & editing. KC: Conceptualization, Data curation, Formal analysis, Investigation, Methodology, Visualization, Writing – review & editing. EW: Data curation, Formal analysis, Investigation, Writing – review & editing. JJ: Conceptualization, Data curation, Formal analysis, Investigation, Methodology, Project administration, Writing – review & editing. JS: Data curation, Writing – review & editing. KR: Formal analysis, Investigation, Visualization, Writing – review & editing. LH: Conceptualization, Formal analysis, Funding acquisition, Methodology, Project administration, Supervision, Visualization, Writing – review & editing. AH: Conceptualization, Data curation, Formal analysis, Funding acquisition, Investigation, Methodology, Project administration, Supervision, Visualization, Writing – review & editing. CS: Data curation, Formal analysis, Investigation, Visualization, Writing – original draft, Writing – review & editing.

## References

[B1] Bell T. M. Shaia C. I. Bearss J. J. Mattix M. E. Koistinen K. A. Honnold S. P.. (2017). Temporal progression of lesions in Guinea pigs infected with lassa virus. Veterinary Pathol. 54 (3), 549–562. doi: 10.1177/0300985816677153 28438110

[B2] Bonwitt J. Sáez A. M. Lamin J. Ansumana R. Dawson M. Buanie J.. (2017). At home with mastomys and rattus: human-rodent interactions and potential for primary transmission of lassa virus in domestic spaces. Am. J. Trop. Med. Hygiene 96 (4), 935–943. doi: 10.4269/ajtmh.16-0675 PMC5392645 28167603

[B3] Brosh-Nissimov T. (2016). Lassa fever: another threat from West Africa. Disaster Military Med. 2, 8. doi: 10.1186/s40696-016-0018-3 PMC5330145 28265442

[B4] Brouwer P. J. M. Antanasijevic A. Ronk A. J. Müller-Kräuter H. Watanabe Y. Claireaux M.. (2022). Lassa virus glycoprotein nanoparticles elicit neutralizing antibody responses and protection. Cell Host Microbe 30 (12), 1759–1772.e12. doi: 10.1016/j.chom.2022.10.018 36400021 PMC9794196

[B5] Cao W. Henry M. D. Borrow P. Yamada H. Elder J. H. Ravkov E. V.. (1998). Identification of alpha-dystroglycan as a receptor for lymphocytic choriomeningitis virus and lassa fever virus. Sci. (New York N.Y.) 282 (5396), 2079–2081. doi: 10.1126/science.282.5396.2079 9851928

[B6] Cashman K. A. Wilkinson E. R. Zeng X. Cardile A. P. Facemire P. R. Bell T. M.. (2018). Immune-mediated systemic vasculitis as the proposed cause of sudden-onset sensorineural hearing loss following lassa virus exposure in cynomolgus macaques. mBio 9 (5), e01896–18. doi: 10.1128/mbio.01896-18 PMC6212830 30377282

[B7] CDC | Bioterrorism Agents/Diseases (2019) Emergency Preparedness & Response. Available at: https://emergency.cdc.gov/agent/agentlist-category.aspprint.

[B8] Childs J. E. Mills J. N. Glass G. E. (1995). Rodent-borne hemorrhagic fever viruses: A special risk for mammalogists? J. Mammalogy 76 (3), 664–680. doi: 10.2307/1382739

[B9] Cohen-Dvashi H. Israeli H. Shani O. Katz A. Diskin R. (2016). Role of LAMP1 binding and pH sensing by the spike complex of Lassa virus. J. Virol. 90 (22), pp.10329–10338. doi: 10.1128/JVI.01624-16 PMC5105667 27605678

[B10] Colangelo P. Verheyen E. Leirs H. Tatard C. Denys C. Dobigny G.. (2013). A mitochondrial phylogeographic scenario for the most widespread african rodent, mastomys natalensis. Biol. J. Linn. Soc. 108 (4), 901–165. doi: 10.1111/bij.12013

[B11] Cross R. W. Hastie K. M. Mire C. E. Robinson J. E. Geisbert T. W. Branco L. M.. (2019). Antibody therapy for lassa fever. Curr. Opin. Virol. 37, 97–104. doi: 10.1016/j.coviro.2019.07.003 31401518

[B12] Cross R. W. Mire C. E. Branco L. M. Geisbert J. B. Rowland M. M. Heinrich M. L.. (2016). Treatment of lassa virus infection in outbred Guinea pigs with first-in-class human monoclonal antibodies. Antiviral Res. 133, 218–222. doi: 10.1016/j.antiviral.2016.08.012 27531367 PMC5032844

[B13] Cummins D. McCormick J. B. Bennett D. Samba J. A. Farrar B. Machin S. J.. (1990). Acute sensorineural deafness in Lassa fever. JAMA 264, 2093–2096. doi: 10.1001/jama.1990.03450160063030 2214077

[B14] Downs I. L. Shaia C. I. Zeng X. Johnson J. C. Hensley L. Saunders D. L.. (2020). Natural history of aerosol induced lassa fever in non-human primates. Viruses 12 (6), 593. doi: 10.3390/v12060593 32485952 PMC7354473

[B15] Eze K. C. Salami T. A. Kpolugbo J. U. (2014). Acute abdominal pain in patients with lassa fever: radiological assessment and diagnostic challenges. Nigerian Med. Journal: J. Nigeria Med. Assoc. 55 (3), 195–2005. doi: 10.4103/0300-1652.132037 PMC4089045 25013248

[B16] Ficenec S. C. Percak J. Arguello S. Bays A. Goba A. Gbakie M.. (2020). Lassa fever-induced hearing loss: the neglected disability of hemorrhagic fever. Int. J. Infect. Diseases: IJID: Off. Publ. Int. Soc. Infect. Dis. 100, 82–87. doi: 10.1016/j.ijid.2020.08.021 PMC7807889 32795603

[B17] Frame J. D. Baldwin J. M. Gocke D. J. Troup J. M. (1970). Lassa fever, a new virus disease of man from West Africa. I. Clinical description and pathological findings. Am. J. Trop. Med. Hygiene 19 (4), 670–676. doi: 10.4269/ajtmh.1970.19.670 4246571

[B18] Garry R. F. (2023). Lassa fever — the road ahead. Nat. Rev. Microbiol. 21 (2), 87–96. doi: 10.1038/s41579-022-00789-8 36097163 PMC9466315

[B19] Günther S. Weisner B. Roth A. Grewing T. Asper M. Drosten C.. (2001). Lassa fever encephalopathy: lassa virus in cerebrospinal fluid but not in serum. J. Infect. Dis. 184 (3), 345–349. doi: 10.1086/322033 11443561

[B20] Happi A. N. Happi C. T. Schoepp R. J. (2019). Lassa fever diagnostics: past, present, and future. Curr. Opin. Virol. 37, 132–138. doi: 10.1016/j.coviro.2019.08.002 31518896 PMC6768724

[B21] Hass M. Gölnitz U. Müller S. Becker-Ziaja B. Günther S. (2004). Replicon system for lassa virus. J. Virol. 78 (24), 13793–18035. doi: 10.1128/jvi.78.24.13793-13803.2004 15564487 PMC533938

[B22] Hensley L. E. Smith M. A. Geisbert J. B. Fritz E. A. Daddario-DiCaprio K. M. Larsen T.. (2011). Pathogenesis of lassa fever in cynomolgus macaques. Virol. J. 8 (1), 2055. doi: 10.1186/1743-422X-8-205 PMC3104370 21548931

[B23] Herring S. Oda J. M. Wagoner J. Kirchmeier D. O'Connor A. Nelson E. A.. (2021). Inhibition of arenaviruses by combinations of orally available approved drugs. Antimicrobial Agents Chemotherapy 65 (4), 10.1128/aac.01146–20. doi: 10.1128/aac.01146-20 PMC8097473 33468464

[B24] HHS and USDA Select Agents and Toxins: 7 CFR Part 331, 9 CFR Part 121, and 42 CFR Part 73. (2023). Available at: https://www.selectagents.gov/sat/list.htm (Accessed August 2023).

[B25] Karnam S. Huang Y. Nguyen N. Yeh S. (2023) Ophthalmic consequences of viral hemorrhagic fevers: insights from the clinic and laboratory.” Front. Trop. Dis. 4 doi: 10.3389/fitd.2023.1107786

[B26] Kayem N. D. Benson C. Aye C. Y. L. Barker S. Tome M. Kennedy S.. (2020). Lassa fever in pregnancy: A systematic review and meta-analysis. Trans. R. Soc. Trop. Med. Hygiene 114 (5), 385–965. doi: 10.1093/trstmh/traa011 PMC7197258 32125412

[B27] Kofman A. Choi M. J. Rollin P. E. (2019). Lassa fever in travelers from West Africa 1969–2016. Emerging Infect. Dis. 25 (2), 236–395. doi: 10.3201/eid2502.180836 PMC6346466 30666924

[B28] Kouadio L. Nowak K. Akoua-Koffi C. Weiss S. Allali B. K. Witkowski P. T.. (2015). Lassa virus in multimammate rats, côte d’Ivoire 2013. Emerging Infect. Dis. 21 (8), 1481–1835. doi: 10.3201/eid2108.150312 PMC4517737 26196447

[B29] Kunz S. Rojek J. M. Perez M. Spiropoulou C. F. Oldstone M. B. A. (2005). Characterization of the interaction of lassa fever virus with its cellular receptor α-dystroglycan. J. Virol. 79 (10), 5979–5875. doi: 10.1128/JVI.79.10.5979-5987.2005 15857984 PMC1091707

[B30] Lassa Fever | CDC (2022). Available at: https://www.cdc.gov/vhf/lassa/index.html.

[B31] Macher A. M. Wolfe M. S. (2006). Historical lassa fever reports and 30-year clinical update. Emerging Infect. Dis. 12 (5), 835–375. doi: 10.3201/eid1205.050052 PMC3374442 16704848

[B32] Malhotra S. Yen J. Y. Honko A. N. Garamszegi S. Caballero I. S. Johnson J. C.. (2013). Transcriptional profiling of the circulating immune response to lassa virus in an aerosol model of exposure. PloS Negl. Trop. Dis. 7 (4), e21715. doi: 10.1371/journal.pntd.0002171 PMC3636129 23638192

[B33] McCormick J. B. Fisher-Hoch S. P. (2002). Lassa fever. Curr. Topics Microbiol. Immunol. 262, 75–109. doi: 10.1007/978-3-642-56029-3_4 11987809

[B34] Mire C. E. Cross R. W. Geisbert J. B. Borisevich V. Agans K. N. Deer D. J. (2017) “Human-monoclonal-antibody therapy protects nonhuman primates against advanced lassa fever.” Nat. Med. 23 (10), 1146–1149 doi: 10.1038/nm.4396 28869611 PMC5719877

[B35] NIH Guidelines for Research Involving Recombinant or Synthetic Nucleic Acid Molecules (NIH Guidelines). (2019). Available at: https://osp.od.nih.gov/wp-content/uploads/NIH_Guidelines.pdf (Accessed August 2023).

[B36] Nwafor C. D. Ilori E. Olayinka A. Ochu C. Olorundare R. Edeh E.. (2021). The one health approach to incident management of the 2019 lassa fever outbreak response in Nigeria. One Health 13, 100346. doi: 10.1016/j.onehlt.2021.100346 34820499 PMC8600060

[B37] Okokhere P. O. Bankole I. A. Akpede G. O. (2013). Central nervous system manifestations of lassa fever in nigeria and the effect on mortality. J. Neurological Sci. 333, e604. doi: 10.1016/j.jns.2013.07.2107

[B38] Price M. E. Fisher-Hoch S. P. Craven R. B. McCormick J. B. (1988). A prospective study of maternal and fetal outcome in acute lassa fever infection during pregnancy. BMJ 297 (6648), 584–587. doi: 10.1136/bmj.297.6648.584 3139220 PMC1834487

[B39] Prioritizing Diseases for Research and Development in Emergency Contexts. (2023). Available at: https://www.who.int/activities/prioritizing-diseases-for-research-anddevelopment-in-emergency-contexts (Accessed August 5, 2023).

[B40] Radoshitzky S. R. de la Torre J. C. (2019). Human pathogenic arenaviruses (Arenaviridae). Encyclopedia Virol. 100, 507–517. doi: 10.1016/B978-0-12-814515-9.00014-X

[B41] Richmond J. K. Baglole D. J. (2003). Lassa fever: epidemiology, clinical features, and social consequences. BMJ: Br. Med. J. 327 (7426), 1271–1275. doi: 10.1136/bmj.327.7426.1271 14644972 PMC286250

[B42] Salam A. P. Duvignaud A. Jaspard M. Malvy D. Carroll M. Tarning J.. (2022). Ribavirin for treating lassa fever: A systematic review of pre-clinical studies and implications for human dosing. PloS Negl. Trop. Dis. 16 (3), e00102895. doi: 10.1371/journal.pntd.0010289 PMC9000057 35353804

[B43] Shehu N. Y. Gomerep S. S. Isa S. E. Iraoyah K. O. Mafuka J. Bitrus N.. (2018). Lassa fever 2016 outbreak in plateau state, nigeria—The changing epidemiology and clinical presentation. Front. Public Health 6. doi: 10.3389/fpubh.2018.00232 PMC6123362 30211144

[B44] Strecker T. Eichler R. Meulen Jt. Weissenhorn W. Dieter Klenk H. Garten W.. (2003). Lassa virus Z protein is a matrix protein and sufficient for the release of virus-like particles [Corrected]. J. Virol. 77 (19), 10700–17055. doi: 10.1128/jvi.77.19.10700-10705.2003 12970458 PMC228511

[B45] White H. A. (1972). Lassa fever: a study of 23 hospital cases. Trans. R. Soc. Trop. Med. Hygiene 66, 390–398. doi: 10.1016/0035-9203(72)90269-6 5046379

