# Supplementary material for: An atlas of gross and histologic lesions and immunohistochemical immunoreactivity during the temporal progression of aerosolized Lassa virus induced hemorrhagic fever in cynomolgus macaques
Source: Front Cell Infect Microbiol. 2024 Feb 9;14:1341891. doi: 10.3389/fcimb.2024.1341891 (PMC10884106; doi:10.3389/fcimb.2024.1341891)
Supplement: Supplementary file 1 [file DataSheet_1.docx]

Supplementary Material

# Supplementary Figures and Tables

## Supplementary Tables

**Supplementary Table 1: Summary of** **histopathologic findings**

Supplementary Table 1a

Supplementary Table 1b

Supplementary Table 1c

Supplementary Table 1d

*early inflammation may be unrelated to LASV

**Supplementary Table 2: Summary of Immunohistochemical results**

Supplementary Table 2a

Supplementary Table 2b

Supplementary Table 2c

Immunoreactivity was graded on the following scale:

0 = none present

r = Rare and scattered, usually single cells

1 = 1-10 cells/hpf

2 = 11-20 cells/hpf

3 = 21-40 cells/hpf

4 = >40 cells/hpf

Note, numbers reflect an estimate of the number of immunoreactive cells per high power field **in foci of immunoreactivity** and not of all the cells in each tissue/organ. For example, in the lungs there may be several high- power fields with only a few immunoreactive cells; however, in foci of inflammation and corresponding immunoreactivity, the number of immunoreactive cells can be much higher. q = tissue not present; n/a = non- applicable

## Supplementary Figures


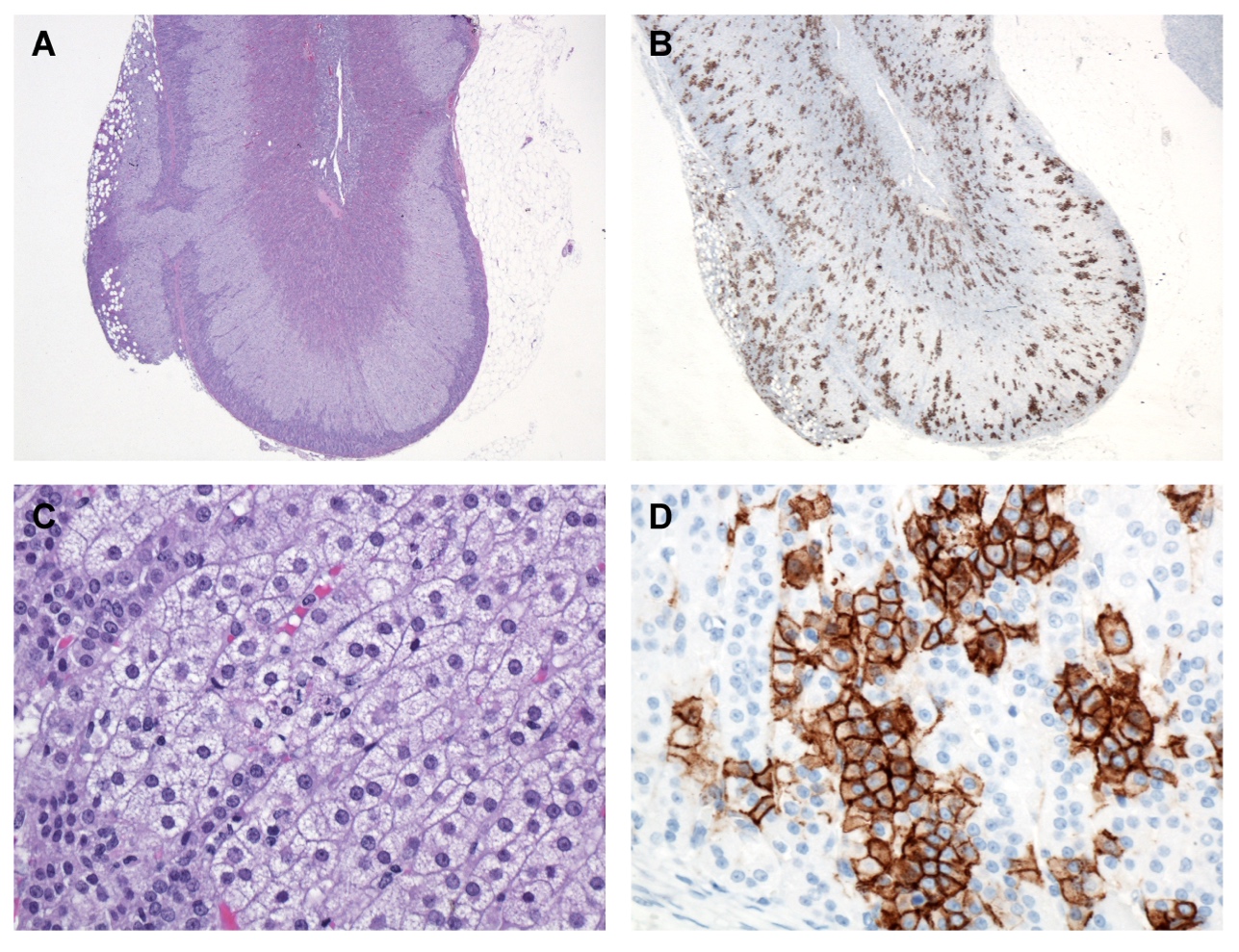


**Supplementary Figure S1**. Adrenal gland, Monkey 18. Day 12 PE. A) The adrenal gland appears normal. HE, 20X. B) Numerous immunoreactive clusters of cortical cells throughout all layers of the cortex. LASV IHC, 20X. C) Higher magnification, demonstrating extremely small foci of necrosis. HE, 400X. D) Higher magnification, demonstrating immunoreactive clusters of cortical cells. LASV IHC, 400X.


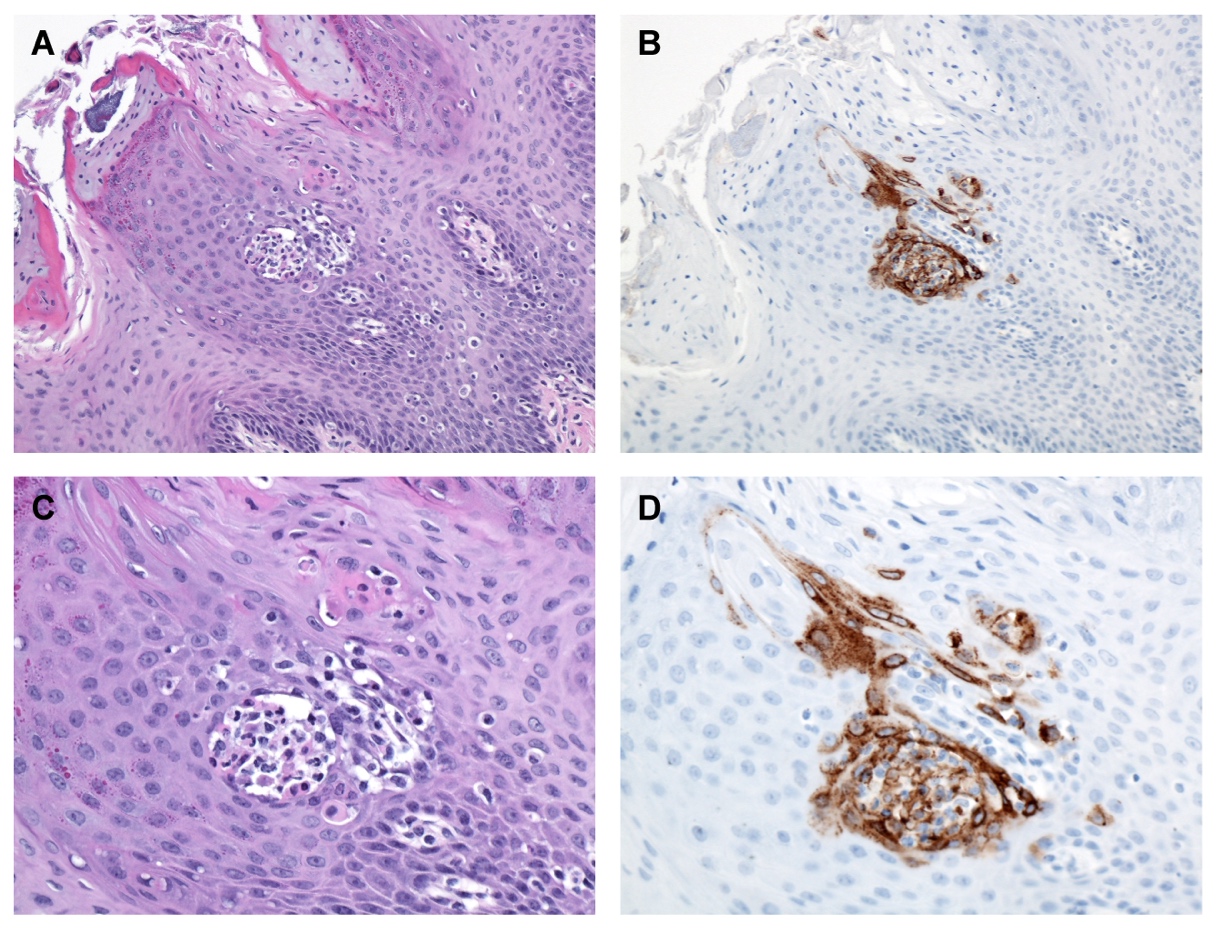


**Supplementary Figure S2**. Tongue, Monkey 11. Day 10 PE. A) Focus of necrosis within the epithelium of the tongue. Note the lack of inflammation. HE, 200X. B) Immunoreactivity focused on area of necrosis. LASV IHC, 200X. C) Higher magnification, demonstrating small focus of necrosis. HE, 400X. D) Higher magnification, demonstrating associated immunoreactivity within the necrotic focus. LASV IHC, 400X.


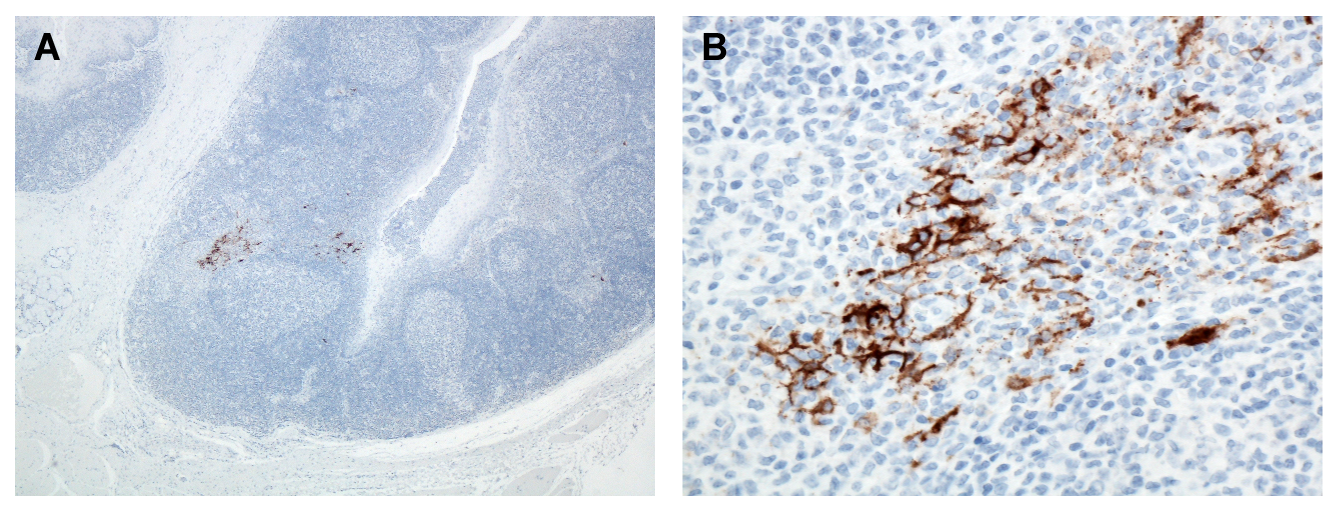


**Supplementary Figure S3**. Tonsil, lymphoid follicles, Monkey 8. Day 6 PE. A) Foci of immunoreactivity of the inter follicular mononuclear cells. LASV IHC, 40X. B) Higher magnification, demonstrating immunoreactive mononuclear cells presumed to be histiocytes or dendritic cells. LASV IHC, 400X. Note, like other lymphoid tissues throughout the body, immunoreactivity is first noted in antigen presenting dendritic cells on or about day 6 PE.


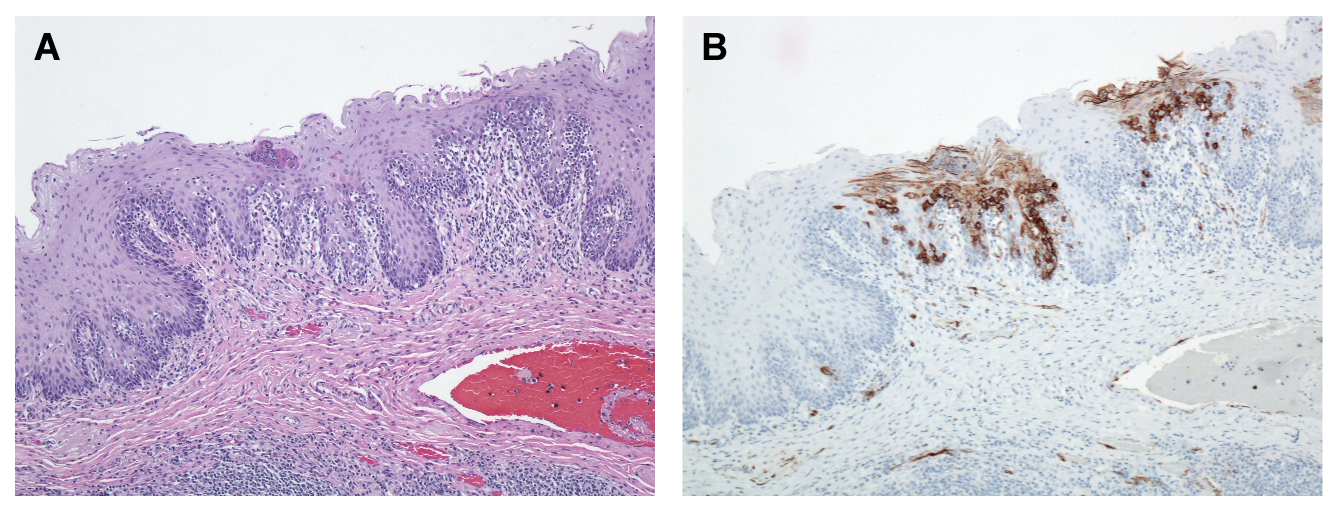


**Supplementary Figure S4**. Tonsil, mucosal epithelium, Monkey 17. Day 12 PE. A) Inflammation and edema at the mucosal-submucosal junction with transmigration of leukocytes. HE, 100X. B) Foci of immunoreactive epithelial cells. LASV IHC, 100X.


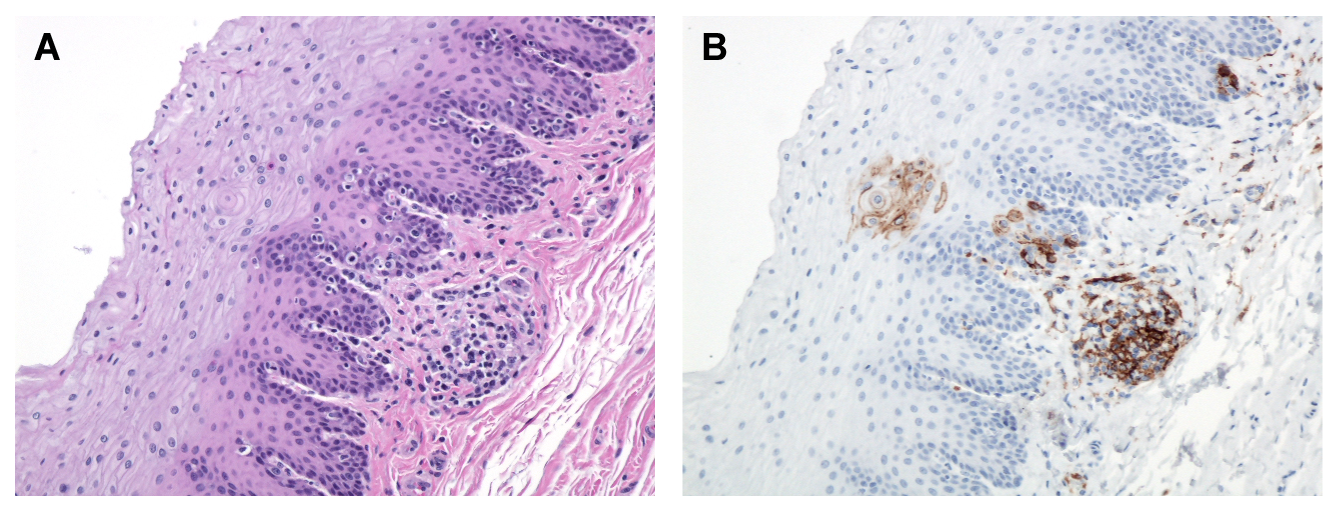


**Supplementary Figure S5**. Esophagus, mucosal epithelium, Monkey 15. Day 11 PE. A) Inflammation at the mucosal-submucosal junction with transmigration of leukocytes. HE, 200X. B) Immunoreactive epithelial and mononuclear inflammatory cells. LASV IHC, 200X.


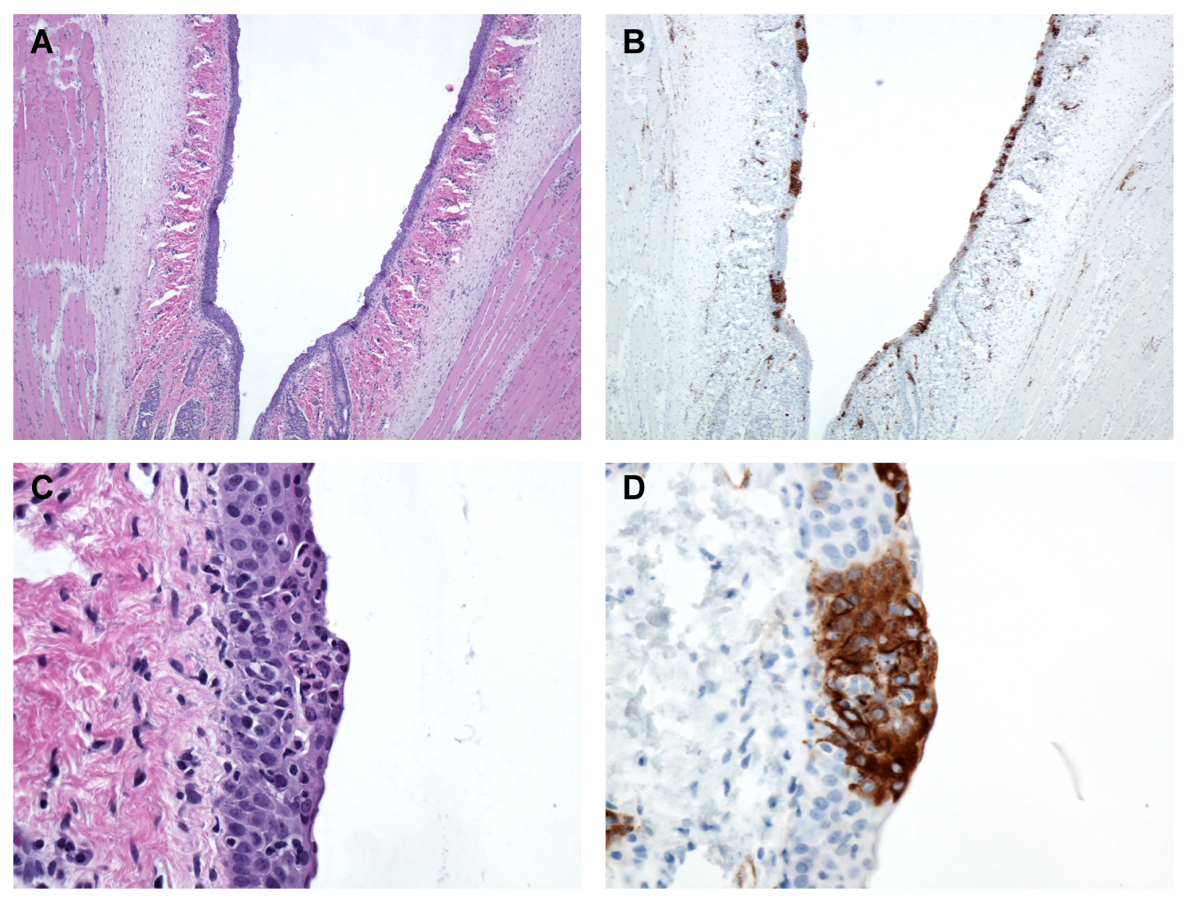


**Supplementary Figure S6**. Larynx, laryngeal epithelium, Monkey 11. Day 10 PE. A) Normal appearing laryngeal epithelium. HE, 40X. B) Multifocal immunoreactivity of the laryngeal epithelium. LASV IHC, 40X C) Higher magnification, demonstrating small focus of epithelial necrosis. HE, 400X. D) Higher magnification, demonstrating associated immunoreactivity within the necrotic focus. LASV IHC, 400X.


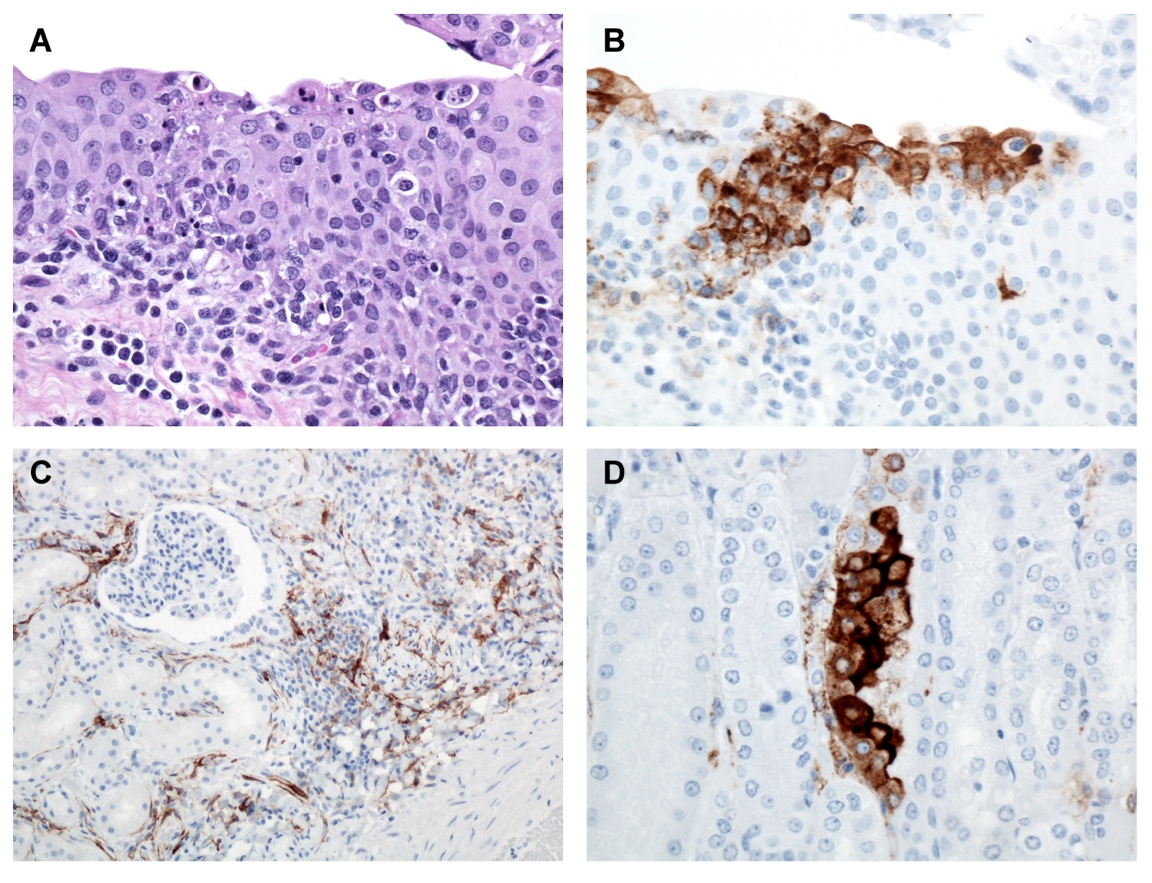


**Supplementary Figure S7**. Kidney, pelvic epithelium, Monkey 11. Day 10 PE. A) Scant inflammation and single cell necrosis of the pelvic epithelium. HE, 400X. B) Multifocal immunoreactivity of the pelvic epithelium. LASV IHC, 400X. C) Interstitial and/or capillary endothelial cell immunoreactivity in a section of cortex. LASV IHC, 200X. D) Renal tubule epithelial cell immunoreactivity, Monkey 16, day 11 PE. LASV IHC, 400X.


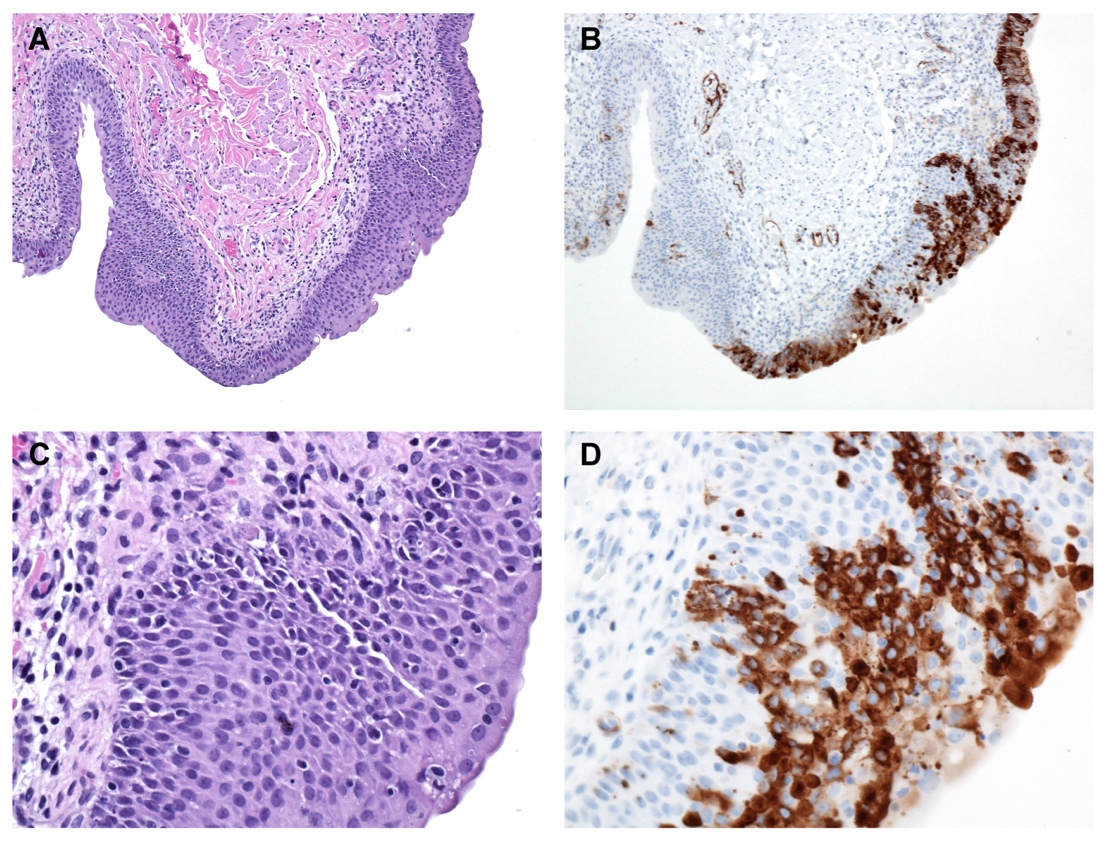


**Supplementary Figure S8**. Urinary bladder, transitional epithelium, Monkey 17. Day 12 PE. A) Normal appearing transitional epithelium with scattered subepithelial inflammation. HE, 100X. B) Multifocal immunoreactivity of the laryngeal epithelium. LASV IHC, 100X. C) Higher magnification, demonstrating degeneration and single cell necrosis. HE, 400X. D) Higher magnification, demonstrating strong cytoplasmic immunoreactivity within the epithelium. LASV IHC, 400X.


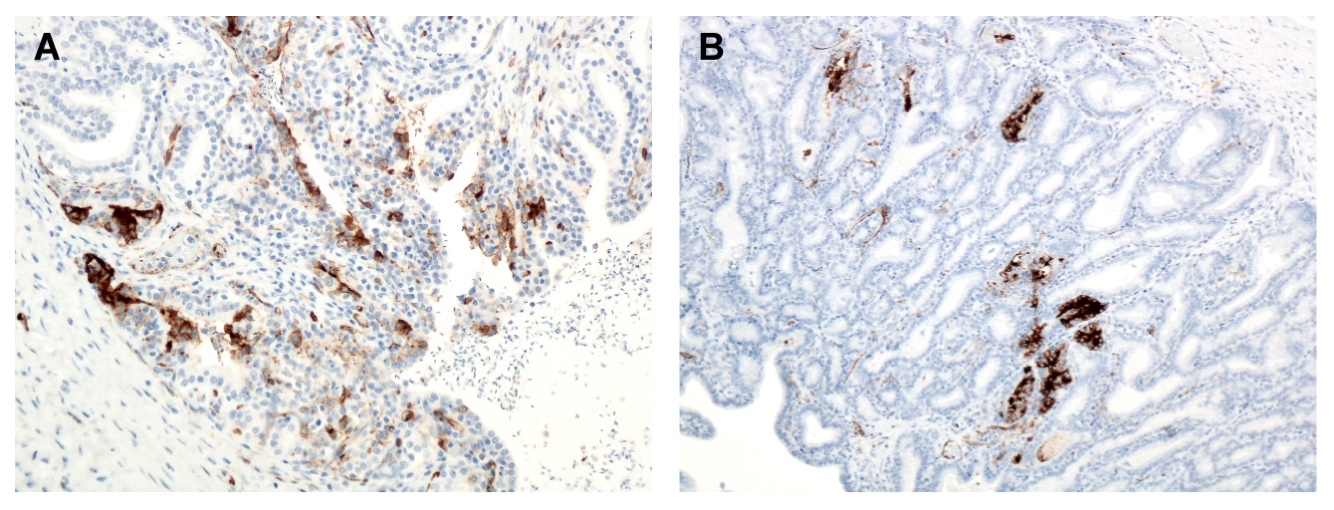


**Supplementary Figure S9**. A) Penile urethral epithelial cell immunoreactivity. Monkey 15 Day 11 PE. LASV IHC, 200X. B) Seminal vesicle glandular epithelial cell immunoreactivity. Monkey 16 Day 11 PE. LASV IHC, 100X.


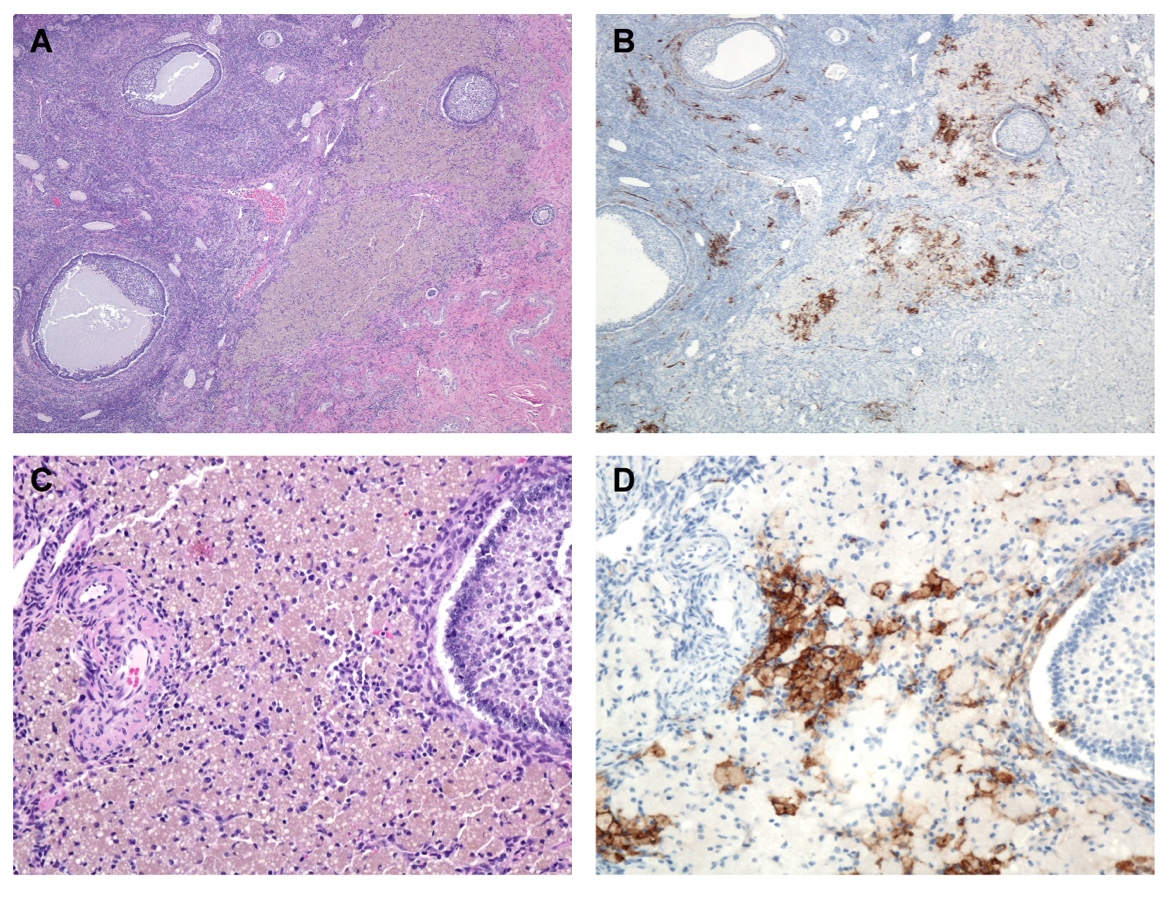


**Supplementary Figure S10**. Ovary, Monkey 19. Day 12 PE. A) Normal appearing developing follicles and luteal tissue. HE, 40X. B) Multifocal immunoreactivity of stromal and luteal cells. LASV IHC, 40X. C) Higher magnification, demonstrating cells of the corpus luteum. HE, 200X. D) Higher magnification, demonstrating strong cytoplasmic immunoreactivity within luteal cells. LASV IHC, 200X.


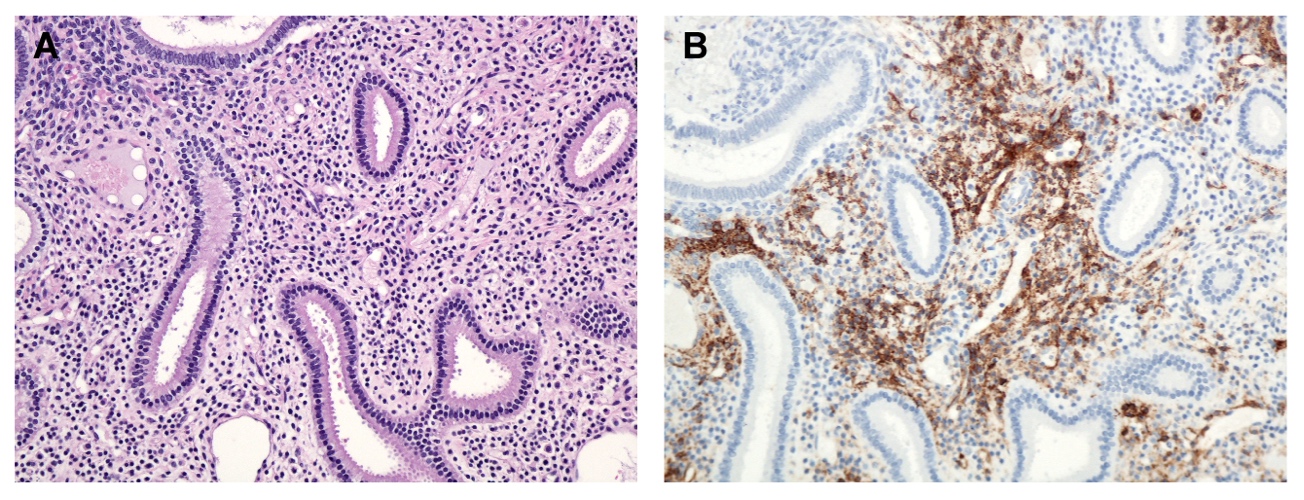


**Supplementary Figure S11**. Uterus, Monkey 19. Day 12 PE. A) Uterine stroma and glands. HE, 200X. B) Multifocal immunoreactivity of uterine stroma. LASV IHC, 200X.


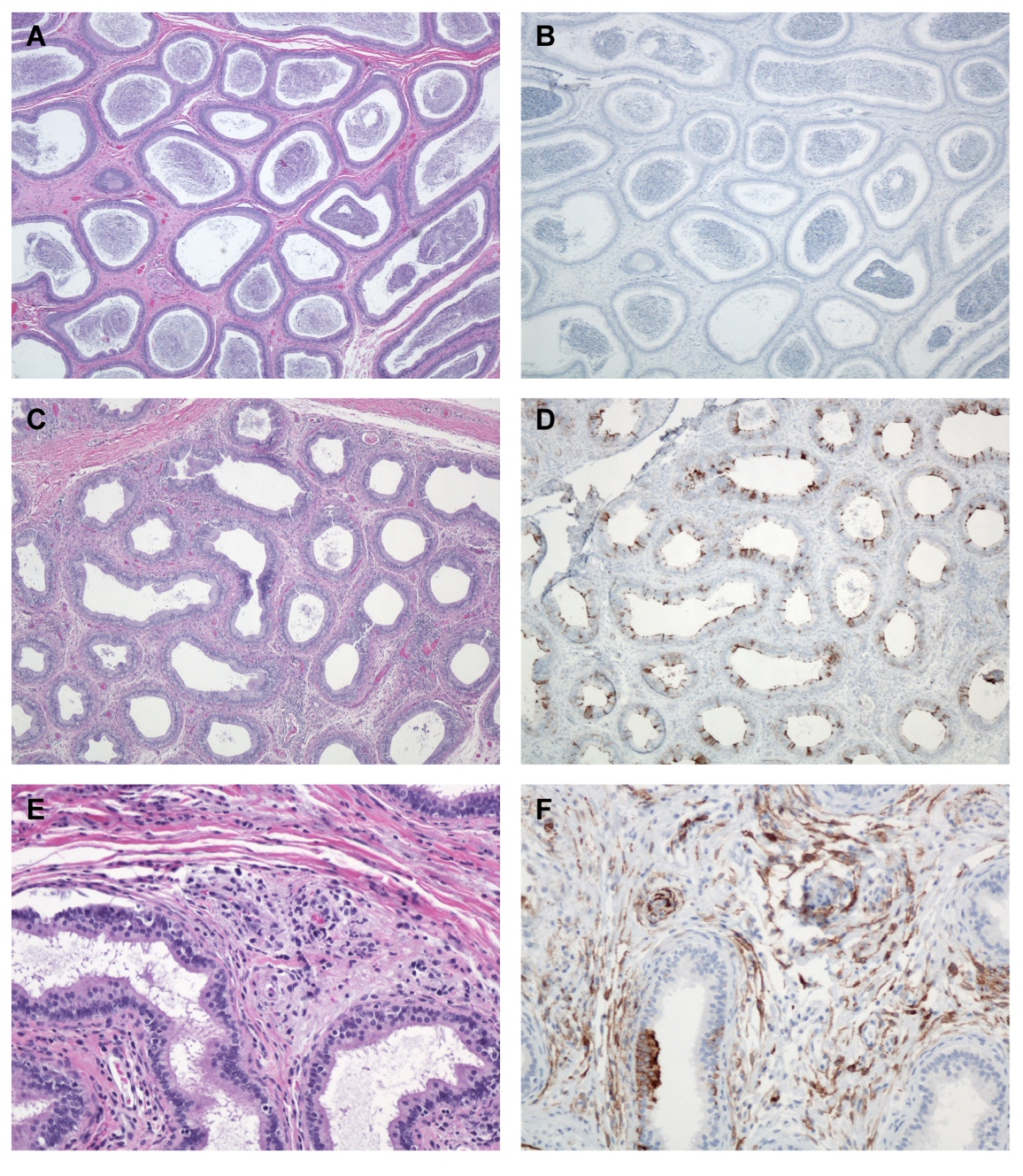


**Supplementary Figure S12**. Epididymis, Monkey 4 Day 3 PE. A) Normal appearing epididymis, tubules and interstitium. HE, 40X. B) Complete lack of LASV immunoreactivity; LASV IHC, 40X. C) Epididymis, Monkey 11 Day 10 PE. HE, 40X. The interstitium is expanded by edema and inflammation and there is a collapsed appearance of the tubule as compared to the day 3 epididymis; D) Widespread LASV immunoreactivity throughout multiple layers of the epididymis; LASV IHC, 40X. E) Higher magnification, demonstrating mononuclear inflammation and edema within the interstitium and distorted tubule lumen. HE, 200X. F) Higher magnification, demonstrating immunoreactivity within the tubule epithelial cells and spindle shaped interstitial cells. LASV IHC, 200X.


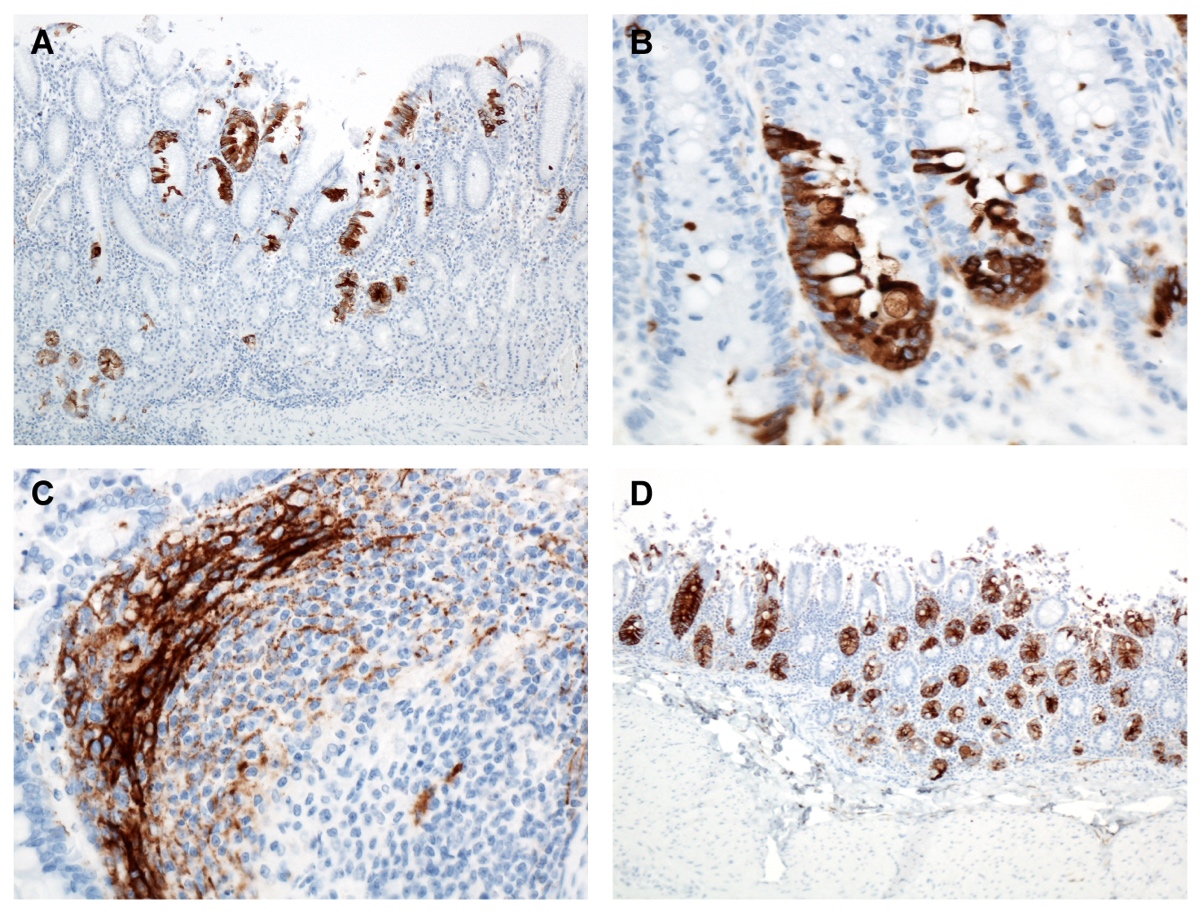


**Supplementary Figure S13**. A) Stomach, gastric mucosal immunoreactivity. Monkey 11. Day 10 PE. LASV IHC, 100X. B) Duodenum, enterocyte immunoreactivity. Monkey 15. Day 11 PE. LASV IHC, 400X. C) Jejunum, immunoreactivity of submucosal GALT. Monkey 18. Day 12 PE. LASV IHC, 400X. D) Ileocecal junction, immunoreactivity of mucosal enterocytes. Monkey 16. Day 11 PE. LASV IHC, 100X.


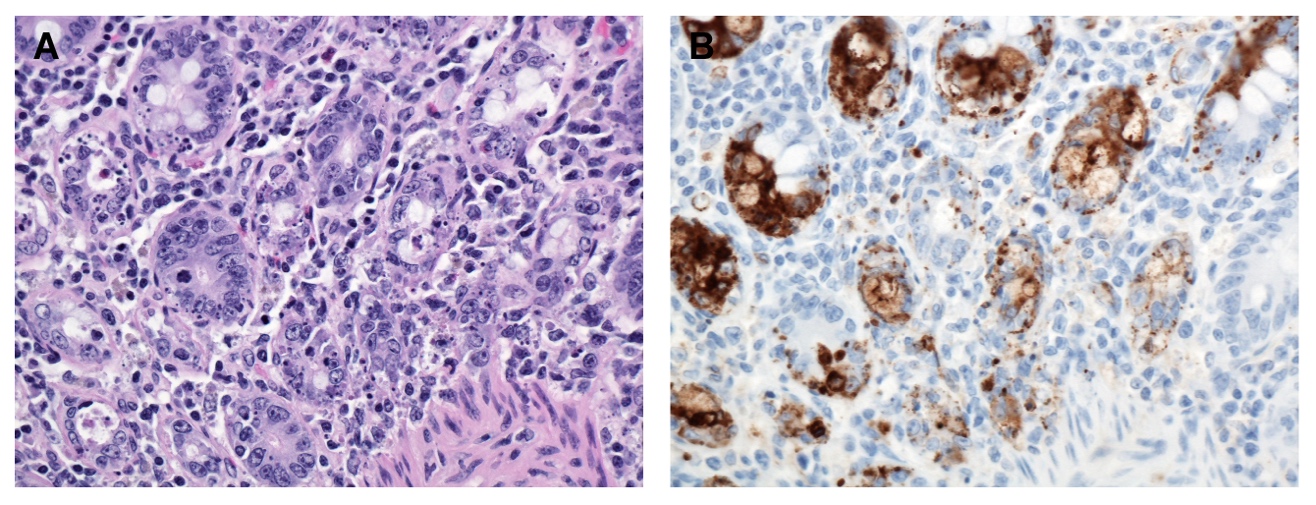


**Supplementary Figure S14**. Colon, Monkey 12. Day 10 PE. A) Mononuclear inflammation and single cell necrosis within the mucosa and lamina propria, HE 200X. B) Colonic mucosal immunoreactivity. LASV IHC, 200X.


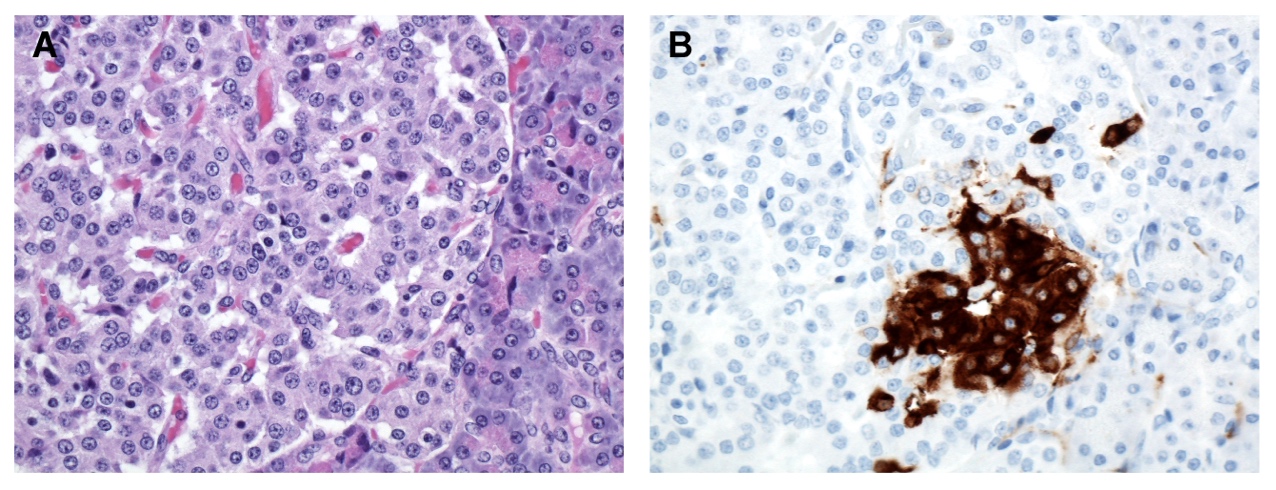


**Supplementary Figure S15**. Pancreas, Islets of Langerhans, Monkey 16. Day 11 PE, A) Normal appearing endocrine (Islets of Langerhans) and exocrine portions of the pancreas, HE 400X. B) Immunoreactivity of Islet cells. LASV IHC, 400X.


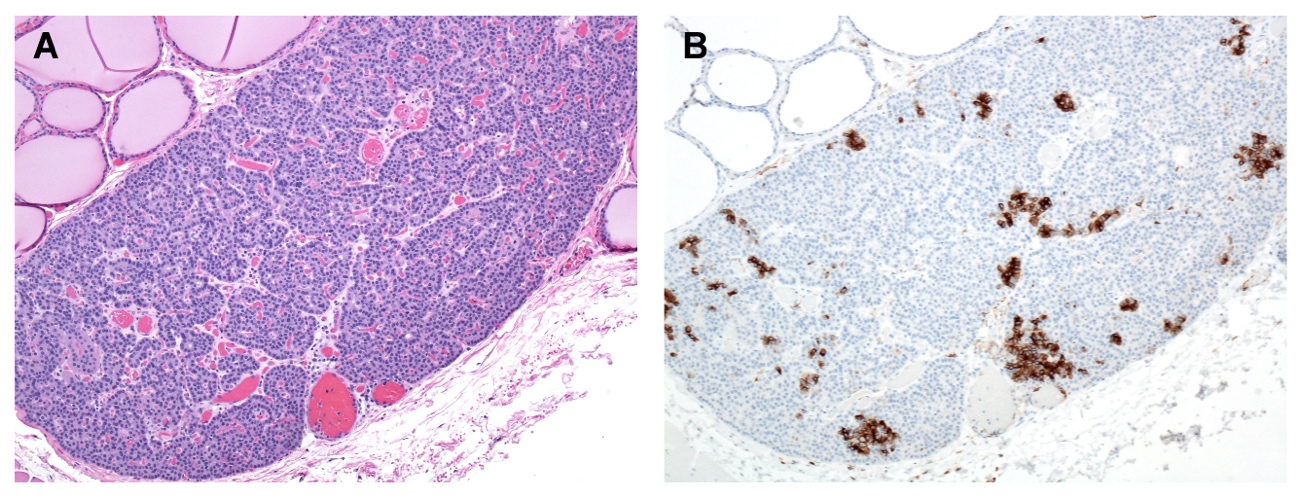


**Supplementary Figure S16**. Parathyroid gland, Monkey 16. Day 11 PE, A) Normal appearing parathyroid gland, HE 100X. B) LASV immunoreactivity of glandular epithelial cells. LASV IHC, 100X.


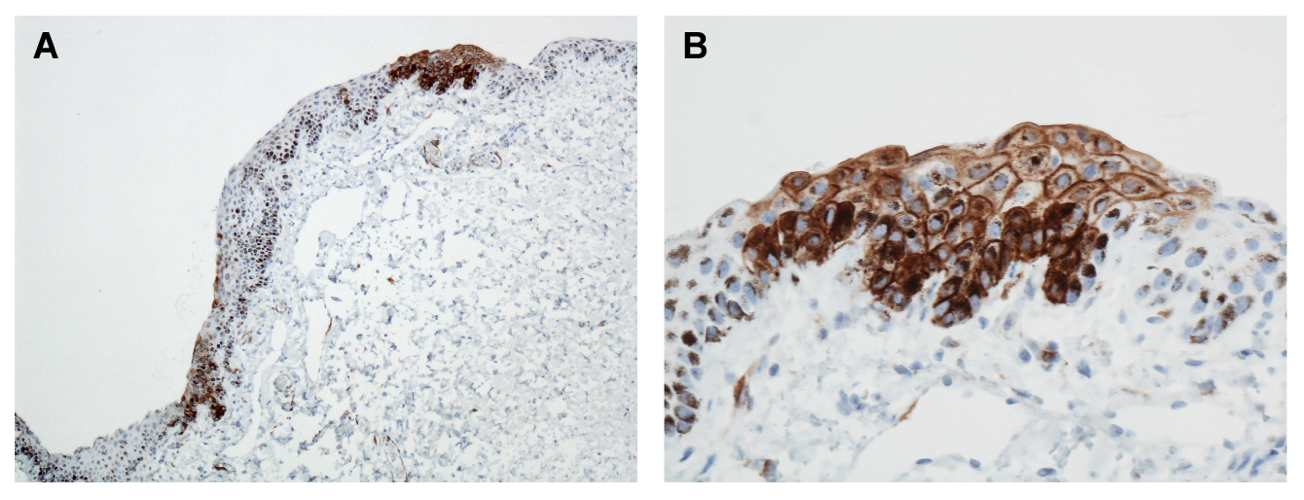


**Supplementary Figure S17**. Eye, conjunctival epithelium, Monkey 17. Day 12 PE. A) Multiple foci of immunoreactivity of the conjunctival epithelium. LASV IHC, 100X. B) Higher magnification demonstrating immunoreactive epithelial cells; LASV IHC, 400X.


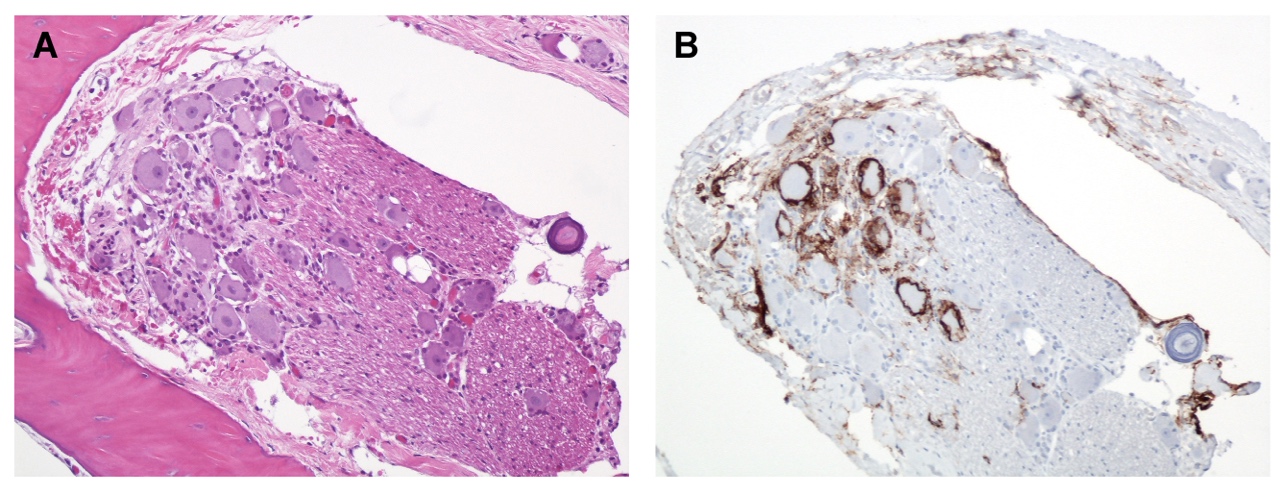


**Supplementary Figure S18**. Ear, vestibulocochlear nerve, Monkey 16. Day 11 PE. A) Nerve and neurons passing through the skull, HE 200X. B) Membrane associated immunoreactivity of neurons of the vestibulocochlear nerve. LASV IHC, 200X.


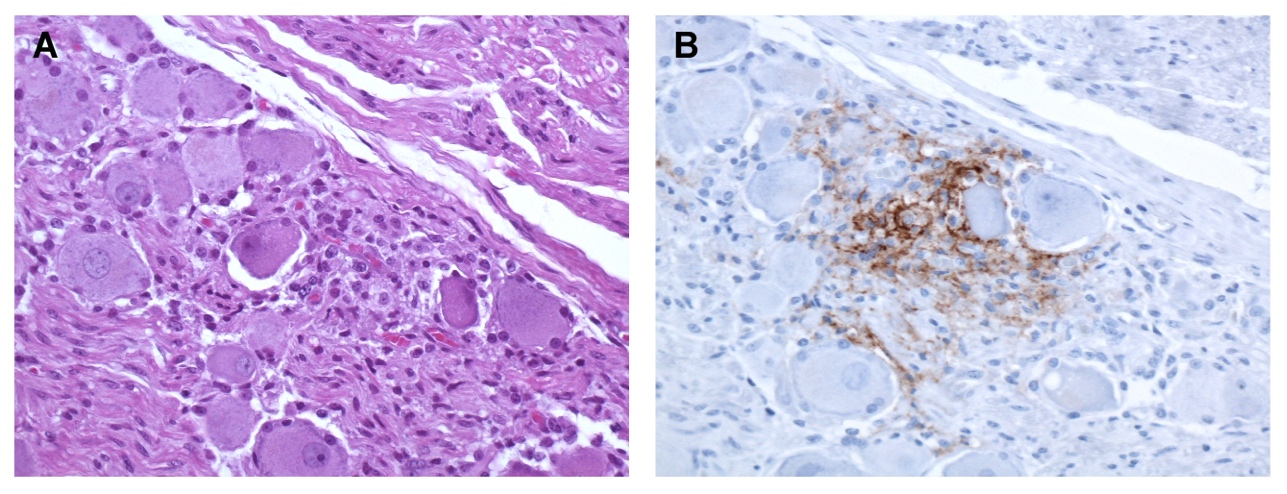


**Supplementary Figure S19**. Ear, vestibulocochlear nerve, Monkey 19. Day 12 PE, A) Nerve and neurons as they pass through the skull. HE 400X. B) Immunoreactivity of neurons and endoneurium of the vestibulocochlear nerve. LASV IHC, 400X.


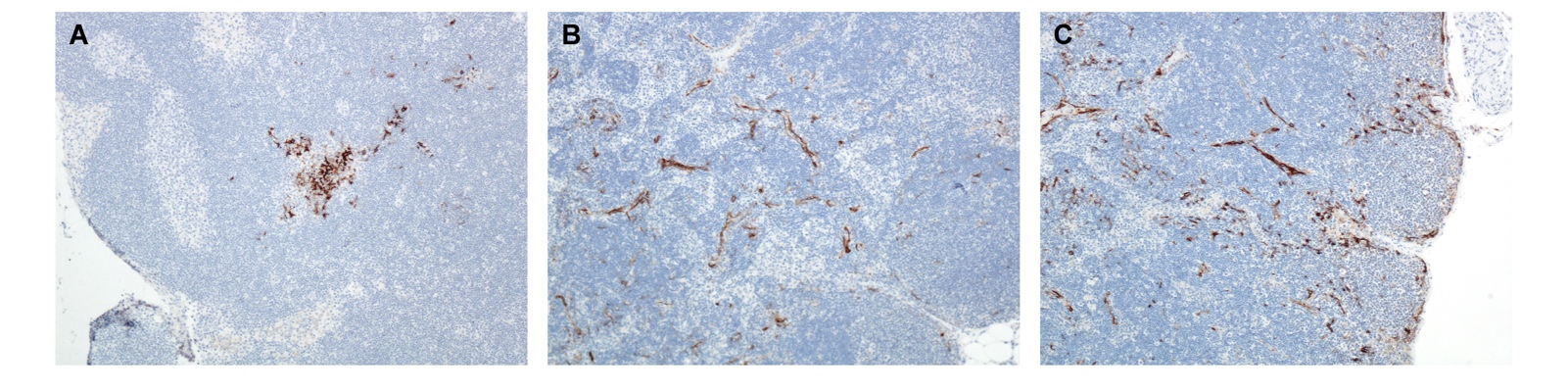


**Supplementary Figure S20**. Mesenteric LN, Monkey 7, Day 6 PE. A) A cluster of immunoreactive sinus mononuclear cells. LASV IHC 100x. B) Mesenteric LN, Monkey 11, Day 10 PE. Immunoreactive sinus endothelial cells. LASV IHC 100x. C) Mesenteric LN, Monkey 15, Day 11 PE. Immunoreactive sinus endothelial cells and scattered mononuclear cells. LASV IHC 100x.


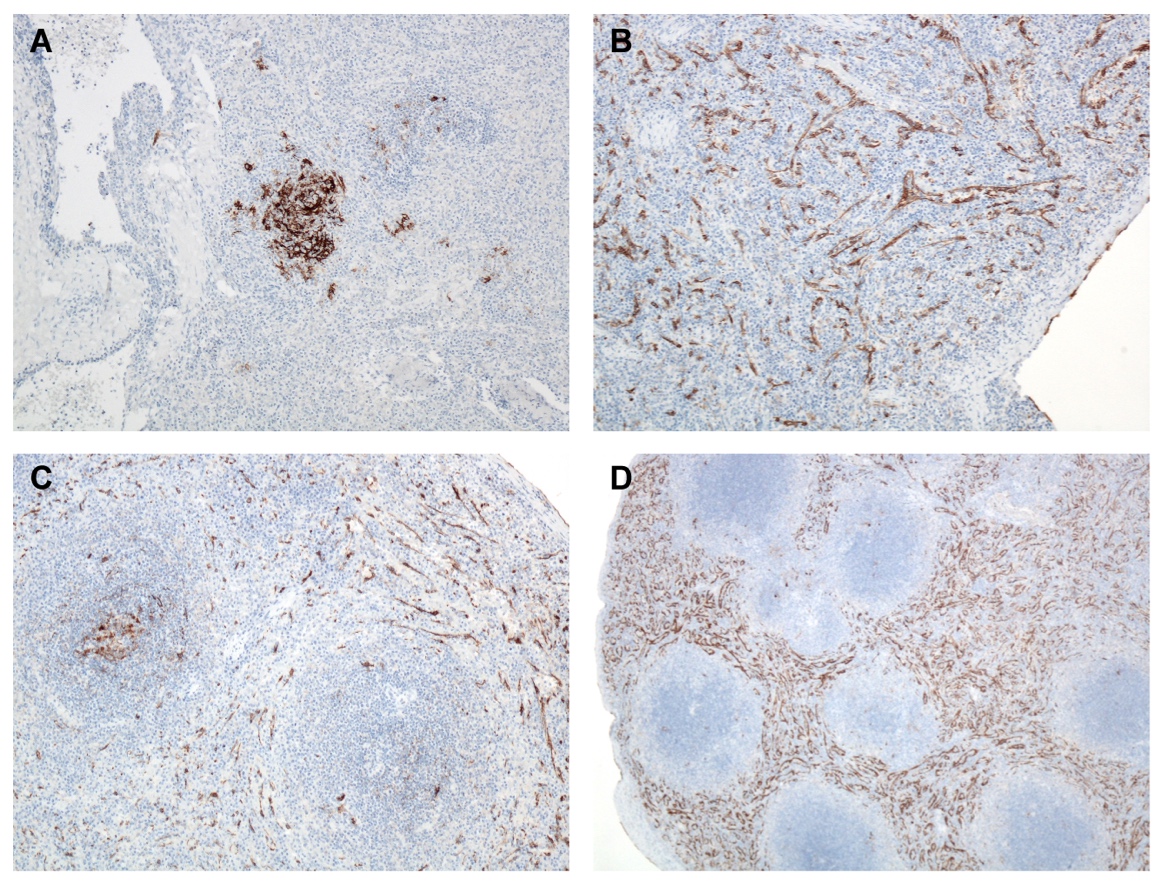


**Supplementary Figure S21**. Spleen, Monkey 7, Day 6 PE. A) A cluster of immunoreactive red pulp mononuclear cells. LASV IHC 100x. B) Spleen, Monkey 11, Day 10 PE. Immunoreactive venous sinus endothelial and fibroblastic reticular cells. LASV IHC 100x. C) Spleen, Monkey 15, Day 11 PE. Immunoreactive venous sinus endothelial cells, scattered mononuclear cells and follicle center immunoreactivity. LASV IHC 100x. D) Spleen, Animal DK9, Day 12 PE. Immunoreactive venous sinus endothelial cells throughout the red pulp. LASV IHC 40x.


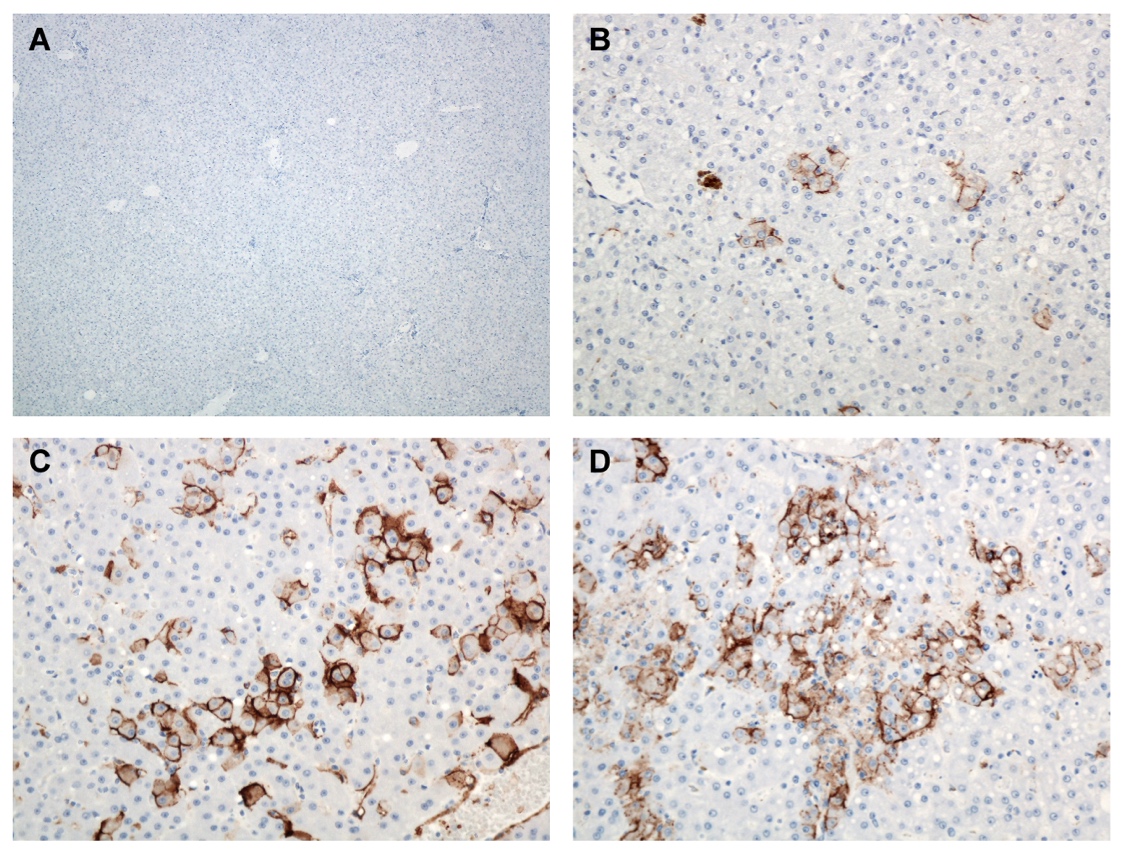


**Supplementary Figure S22**. Liver, Monkey 9, Day 6 PE. A) Complete lack of immunoreactivity. LASV IHC 40x. B) Liver, Monkey 12, Day 10 PE. Membrane associated immunoreactivity of individual and small clusters of hepatocytes. LASV IHC 200x. C) Liver, Monkey 15, Day 11 PE. Increased number of immunoreactive hepatocytes and Kupffer cells within sinusoids. LASV IHC 200x. D) Liver, Monkey 18, Day 12 PE. Immunoreactive debris corresponds to a focus of necrosis. LASV IHC 200x.


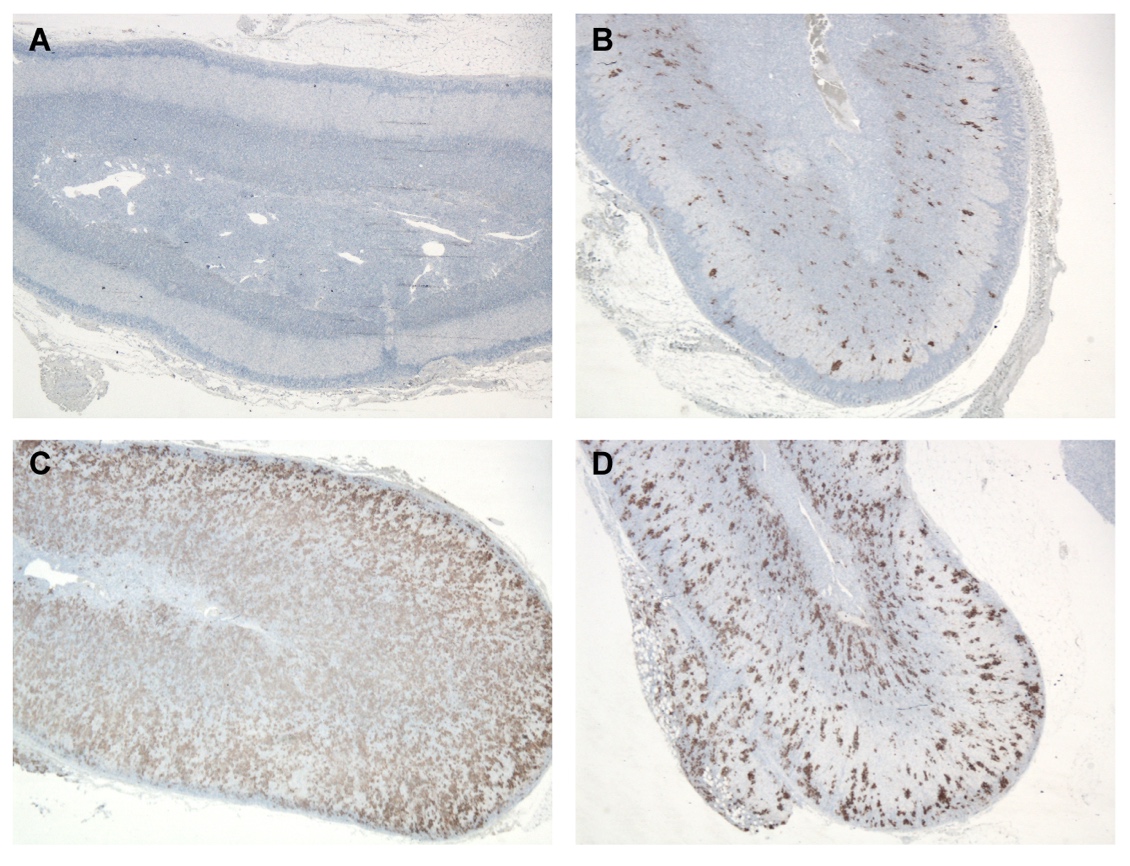


**Supplementary Figure S23**. Adrenal gland, Monkey 5, Day 6 PE. A) Complete lack of immunoreactivity. LASV IHC 20x. B) Adrenal gland, Monkey 12, Day 10 PE. Immunoreactivity of individual and small clusters of cells throughout the cortex. LASV IHC 20x. C) Adrenal gland, Monkey 16, Day 11 PE. Diffuse immunoreactivity of the cortex. LASV IHC 20x. D) Adrenal gland, Monkey 18, Day 12 PE. multifocal epithelial cell immunoreactivity throughout the cortex. LASV IHC 20x.
